# The minipig intraoral dental implant model: A systematic review and meta-analysis

**DOI:** 10.1371/journal.pone.0264475

**Published:** 2022-02-28

**Authors:** Marta Liliana Musskopf, Amanda Finger Stadler, Ulf ME Wikesjö, Cristiano Susin

**Affiliations:** Division of Comprehensive Oral Health–Periodontology, Laboratory for Applied Periodontal & Craniofacial Research, Adams School of Dentistry, University of North Carolina at Chapel Hill, Chapel Hill, NC, United States of America; Ohio State University, UNITED STATES

## Abstract

**Objectives:**

The objective of this report was to provide a review of the minipig intraoral dental implant model including a meta-analysis to estimate osseointegration and crestal bone remodeling.

**Methods:**

A systematic review including PubMed and EMBASE databases through June 2021 was conducted. Two independent examiners screened titles/abstracts and selected full-text articles. Studies evaluating titanium dental implant osseointegration in native alveolar bone were included. A quality assessment of reporting was performed. Random-effects meta-analyses and meta-regressions were produced for bone-implant contact (BIC), first BIC, and crestal bone level.

**Results:**

125 out of 249 full-text articles were reviewed, 55 original studies were included. Quality of reporting was generally low, omissions included animal characteristics, examiner masking/calibration, and sample size calculation. The typical minipig model protocol included surgical extraction of the mandibular premolars and first molar, 12±4 wks post-extraction healing, placement of three narrow regular length dental implants per jaw quadrant, submerged implant healing and 8 wks of osseointegration. Approximately 90% of studies reported undecalcified incandescent light microscopy histometrics. Overall, mean BIC was 59.88% (95%CI: 57.43–62.33). BIC increased significantly over time (p<0.001): 40.93 (95%CI: 34.95–46.90) at 2 wks, 58.37% (95%CI: 54.38–62.36) at 4 wks, and 66.33% (95%CI: 63.45–69.21) beyond 4 wks. Variability among studies was mainly explained by differences in observation interval post-extraction and post-implant placement, and implant surface. Heterogeneity was high for all studies (I^2^ > 90%, p<0.001).

**Conclusions:**

The minipig intraoral dental implant model appears to effectively demonstrate osseointegration and alveolar bone remodeling similar to that observed in humans and canine models.

## Introduction

Per-Ingvar Brånemark studying micro-circulation using a rodent model fortuitously discovered that devices made from titanium while biocompatible also formed an intimate relationship with adjoining bone [1]. This initial discovery was confirmed in humans and every year millions of patients benefit from titanium dental implant-anchored prosthetic rehabilitations. Animal models have been used extensively to study soft and hard tissue responses to dental implant materials and designs over the last 50 years [2]. Thousands of animal studies have been published reporting on novel implant technologies, surgical techniques, and alveolar bone augmentation strategies. The use of rodent models and extra-oral sites in large animal models provide insights into the biology of osseointegration and represent useful screening tools of new designs and technologies; however, they fail to mimic the complexity of the oral environment and uniqueness of the alveolar bone. Only large animal intraoral models allow the use of clinically relevant dental implants and prosthetic components.

Historically, canine and nonhuman primate platforms have been preferred for oral/maxillofacial research, however porcine/minipig models have emerged as an important alternative [3,4]. The minipig has been widely used in biomedical research including cardiovascular, orthopedic, and dermatologic settings due to similarities with humans in the anatomy and physiology [5]. Regarding the oral cavity, minipigs feature deciduous, mixed, and permanent dentitions; the first permanent molar is the first permanent tooth to erupt, and there is an extended mixed dentition period. Whereas the minipig and humans share tooth types, the minipig features 6 maxillary and mandibular incisors rather than 4, and 8 maxillary/mandibular premolars rather than 4. Periodontally healthy minipigs feature shallow to moderate probing depths [3]. Keratinized tissue width averages 2.7±0.8mm [6]. Minipig and humans have similar bone formation and remodeling rates [7]. Pilawski et al. (2021) compared maxillary alveolar bone structure in minipigs and humans using radiography, histology, and immunohistochemistry [8]. Histologically, the collagen organization, osteocyte density, alveolar bone remodeling, and mineral apposition rate were similar. Radiographically, bone architecture, bone mineral density, and bone volume were also comparable [8]. Bone formation in gap defects has been estimated to be 1.2–1.5mm per day in minipigs and 1.0–1.5mm per day in humans [2].

Herein, we report a systematic review and meta-analysis of a minipig intraoral dental implant model used to evaluate dental implant technologies and study peri-implant tissue healing. Histological observations from minipig, canine and human studies are discussed in a clinical perspective.

## Methods

The Preferred Reporting Items for Systematic Reviews and Meta-Analysis (PRISMA) was followed during the review process and reporting [9].

### Focused questions

The literature was systematically searched to answer the following focused questions:

- What are the osseointegration and crestal bone remodeling levels in the minipig intraoral dental implant model?
- Which factors explain the different results observed in the literature?

### Search strategy

An electronic search of MEDLINE (via PubMed) and EMBASE up to June 2021 was conducted using the following combination of MeSH terms:

For PubMed

((((dental implant[MeSH Terms]) OR (dental implantation[MeSH Terms])) OR (tooth implantation[MeSH Terms])) AND ((miniature swine[MeSH Terms]) OR (miniature pig[MeSH Terms]) OR (micropig)))

For EMBASE

(’minipig’ OR ’miniature swine’ OR ’mini pig’ OR ’miniature pig’ OR ’micropig’) AND (’tooth implant’ OR ’dental implant’ OR ’tooth implantation’) AND [embase]/lim

A manual search of the list of references of the included studies was performed. No efforts were undertaken to search the grey literature.

### Study selection

Original articles using minipigs, intraoral sites, titanium dental implants, and evaluating osseointegration histologically were included. Publications without proper statistical analysis including central tendency measures (means or medians) and variability (standard deviation or data range) were excluded from the analysis.

#### Animals’ characteristics

Only studies with data of systemically healthy animals were included. For those studies that also included animals with systemic diseases/conditions, only data from healthy controls were used.

#### Type of treatments

Only data derived from implants placed in native bone were included. For studies that placed implants in augmented bone or that carried out implant placement concomitantly with guided bone regeneration, only data from control groups were used.

### Outcomes

The primary outcome of interest was bone-implant contact (BIC). Secondary outcomes were distance between the implant platform and the first bone-implant contact (first BIC) and distance between the implant platform and the crestal alveolar bone. Osseointegration was defined as the percentage of BIC measured along the length of the implant within the extension of alveolar bone/total perimeter of the implant. First BIC was defined as the distance between the most coronal BIC and the implant platform. Crestal bone level/loss was defined as the distance between the most coronal extent of crestal bone along the implant and the implant platform.

### Data synthesis

Two reviewers (MLM and AFS) independently screened titles and abstracts through the databases. Any disagreement was solved by consensus between the reviewers or by a third reviewer (CS). One examiner (MLM) extracted data from the selected studies, and data was reviewed for completeness and accuracy (CS).

### Studies characteristics and quality of reporting

Studies characteristics, including sample, preparatory and implant placement protocols, histology performed, and main findings are summarized in table format. Quality assessment of the studies included in the meta-analyses was evaluated based on selected items from ARRIVE checklist (see S1 Table) [10].

### Statistical analysis

Meta-analyses were performed for histological parameters for which data could be extracted from at least 3 studies. Articles reporting means and standard deviations were included in the meta-analysis. Studies that only reported medians, data range and sample size were also included, and means and standard deviations were calculated using appropriate formulas [11]. Studies that only presented results in graphic format were not included. Data analysis was performed using statistical software (Stata 17 for Mac, Stata Corporation, College Station, TX, USA). Random effects models were used to estimate the effect sizes and 95% confidence intervals (CI) [12]. Random-effects meta-regression analysis was carried out to investigate factors (moderators) that could help explain between-study heterogeneity. Animal strain and age, healing after extraction and implant placement, staging, type of healing, loading and implant surface were considered. The restricted maximum likelihood method was used. The heterogeneity of effects among studies was assessed by calculating I^2^ and was broadly categorized as low, moderate and high following the I^2^ statistics cut-off points suggested by Higgins et al. (2003): 25%, 50%, and 75% [13]. Publication bias was investigate using funnel plots and Egger’s test for funnel plot asymmetry [14]. Exploratory analyses investigating quality of reporting and study funding were done. A total score for quality of reporting was generated by adding scores for each item as follows: 0 = not reported; 1 = unclear; 2 = reported. Funding was categorized as public, private, combined public and private, and unclear.

Ethics approval was not required for this systematic review and meta-analysis.

## Results

### Studies selection and characteristics

**Fig 1:**
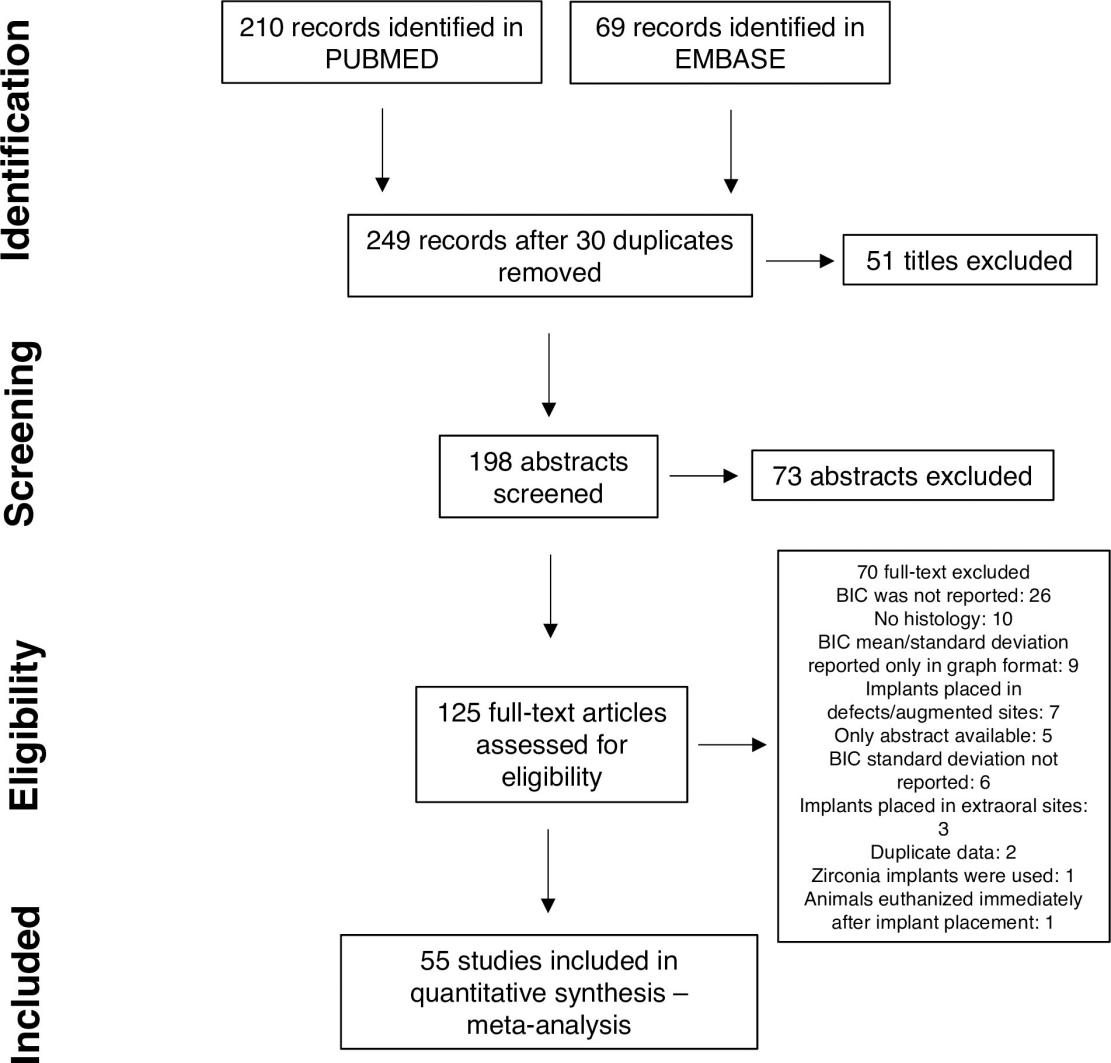
Flowchart describing the study selection process.

The bibliographic search yielded 279 publications (Fig 1) and a clear increase in the number of articles published overtime was observed. Agreement between reviewers was 85% for titles and 80% for abstracts selection. Fifty-five studies [15–69] were included in the quantitative analysis and the most frequent reason for exclusion from the review was lack of BIC data (47.27% of studies) (S2 Table). No additional studies were found in the reference list of studies included.

Studies are summarized in S3 Table. Most studies focused on evaluating new implant surface technologies (47.27%), implant material (10.91%), implant design (7.27%), and surgical protocol (7.27%). The minipig strain most used was the Göttingen (30.90%), followed by Lanyu small-ear pigs (7.27%). The animal’s age ranged from 12 to 72 months and the weight average was 48.99±5.57 kg. Most studies used only females (49.09%). The average number of animals included in the studies was 10.10±5.57 (range 3–30).

Premolars and first molars were usually extracted to provide space for posterior implant placement; few studies extracted incisors or placed implants in diastemas. Immediate implant placement occurred in only 5 studies (9.09%). For delayed implant placement studies, healing following extractions ranged between 8 and 32 wks; most studies allowed for 12 wks of healing post-extraction (36.36%). The average number of implants placed per animal was 6.49±3.63 (range: 2–20), and most studies placed implants in the mandible only (64%). Most studies used implants with 3.5mm in diameter (range: 3.3–6.0mm) and 8mm in length (range: 5-15mm). The average healing time following implant placement was 8.87±10.76 wks (range: 1–96). Delayed implant placement and submerged healing were used in 80% and 64% of studies, respectively. Transmucosal healing was used in 20 out of 55 (36.4%) studies; 14 out of 20 (70%) studies used healing abutments or stock abutments/healing caps. Four (20%) studies used stock abutments and provisional restorations, and two (10%) studies used stock abutments and metallic/ceramic crowns. Approximately 60% of studies reported the use of antibiotics following implant placement. Chemical plaque control was reported by 2 (3.64%) studies and in 4 (7.27%) studies a professional dental cleaning was performed during the follow up time.

All studies used the cutting-grinding technique for histologic preparation of undecalcified samples and 90% used light microscopy for histological evaluation. A buccal-lingual orientation was used in 55% of the studies and section thickness ≥50μm was used in 51% of the studies (range: 50-150μm) when light microscopy was used. Only 15 studies (27.3%) reported that more than one section was used for histological analysis. Toluidine blue staining was used in 45% of studies.

### Quality of reporting

**Fig 2:**
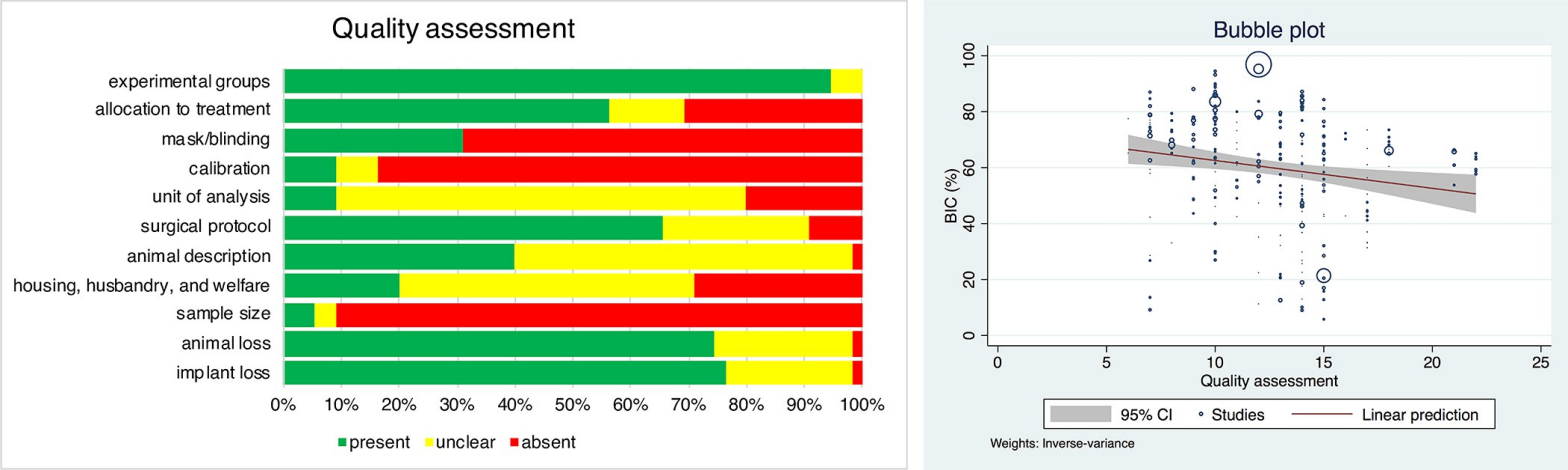
a. Quality assessment of the 55 studies included in the systematic review. b. Bubble plot of BIC% and quality assessment scores.

Reporting of selected items from the ARRIVE checklist are presented in Fig 2 and S4 Table. Fig 2A presents the distribution of the abovementioned items for the selected studies. Overall, 94.54% of studies described the experimental groups, 74.55% reported animal loss, 56.36% allocated treatment using randomization, and 65.45% described the surgical protocol for implant placement. Most studies described the surgical protocol for implant placement as following the manufacturer’s protocol. Details of animal used were fully described by 40% of the studies. Low quality was related to absence of sample size calculation (94.54% of studies), calibration (83.64% of studies), and masking/blinding (69.09% of studies). Implant loss, an important adverse event, was reported in 76.36% of studies, ranging between 0 and 47 implants, and on average 6.29±11.42 were reported lost.

### Primary outcome

**Fig 3:**
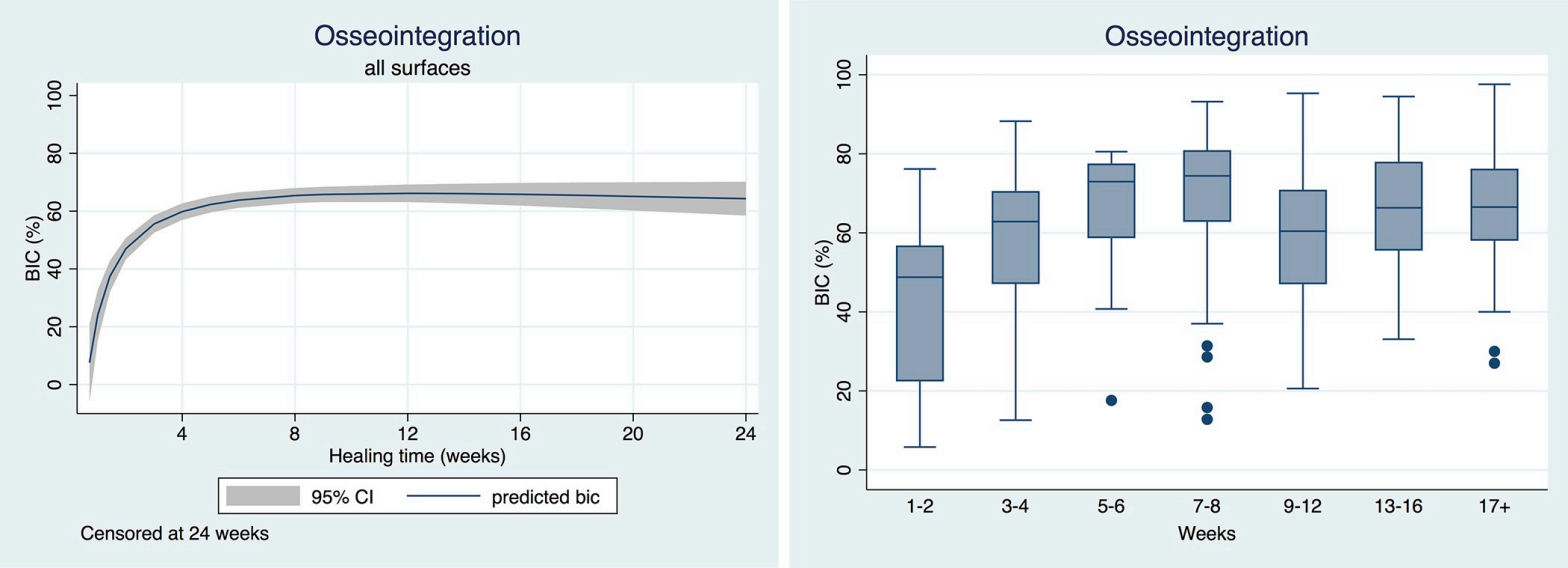
a. Predicted bone-implant contact (BIC) over time. b. Box plot of bone-implant contact (BIC) according to healing time.

Table 1 presents BIC according to healing period. Overall, BIC was 59.88% (95%CI: 57.43–62.33). BIC increased significantly during the first month of healing levelling off afterwards (Fig 3A). A high degree of variability was observed in each healing period (Fig 3B). Meta-regressions were used to explore between-study heterogeneity, and crude and adjusted BIC estimates are presented according to important covariates in Table 2. In the unadjusted analysis, between-study heterogeneity could be explained by animal age, alveolar ridge healing time, immediate implant placement, implant loading, implant healing time, and implant surface.

**Table 1:** Osseointegration (BIC %) according to observation interval (wks).

Observation interval	Mean	95%CI		I^2^	p-value
1–2	40.93	34.95	46.90	97.43	<0.001
3–4	58.37	54.38	62.36	94.59	<0.001
5–6	65.79	58.53	73.05	96.63	<0.001
7–8	69.13	64.82	73.43	98.19	<0.001
9–12	58.49	48.64	68.34	99.39	<0.001
13–16	68.32	60.05	76.58	96.60	<0.001
17	64.33	58.33	70.34	97.51	<0.001
Overall	59.88	57.43	62.33	98.90	<0.001

BIC: Bone-implant contact; CI: Confidence interval; I^2^: Heterogeneity index.

**Table 2:** Unadjusted and adjusted osseointegration (BIC %) estimates according to covariates (meta-regression).

		Crude			Adjusted		
		Mean	95%CI		Mean	95%CI	
Age	<18 months	64.96A	61.13	68.78			
	18–24 months	58.62AB	53.53	63.71			
	>24 months	56.90B	51.84	61.95			
Arch	Maxilla	60.21A	56.40	64.03			
	Mandible	60.21A	56.99	63.43			
	Maxilla/Mandible	52.78A	37.20	68.37			
Ridge healing	≤8 wks	56.89A	51.81	61.97	55.90A	51.49	60.31
	>8 - ≤12 wks	59.26A	55.27	63.25	58.41A	55.13	61.69
	>12 wks	63.27B	59.62	66.92	65.84B	61.96	69.71
Immediate	Delayed	61.29A	58.82	63.76			
	Immediate	39.21B	30.79	47.63			
Staging	Submerged	58.77A	55.85	61.69			
	Mixed	61.99A	52.68	71.30			
	Non-submerged	66.24A	62.03	70.44			
Loading	No	59.21A	56.62	61.80			
	1–12 wks	70.94AB	67.38	74.49			
	>12 wks	74.44B	67.28	81.60			
Implant healing	≤2 wks	40.93A	34.95	46.90	37.50A	32.09	42.91
	>2 - ≤4 wks	58.37B	54.38	62.36	58.42B	54.45	62.40
	≥5 wks	66.33C	63.45	69.21	67.23C	64.39	70.08
Surface	Machined	54.04A	41.82	66.25	56.75AB	47.09	66.40
	Coated	54.04A	49.10	58.99	53.59A	48.50	58.68
	Mod rough/not SLA	60.63A	57.04	64.21	58.74A	55.47	62.00
	SLA	62.83B	58.36	67.29	65.32BC	61.69	68.95

BIC: Bone-implant contact; CI: Confidence interval; I^2^: Heterogeneity index; I^2^ > 90% for all models; estimates followed by the same capital letters did not differ significantly (p>0.05).

SLA: Sandblasted acid-etched.

In the adjusted analysis, healing time following extractions, healing time after implant placement, and implant surface remained statistically significant factors. Studies that used more than 12 wks of healing following extraction and more than 5 wks of healing after implant placement had significantly higher BIC. Studies testing implants with SLA surface had significantly higher BIC than studies testing other surfaces. An exploratory meta-regression showed an inverse relationship between quality of reporting and BIC (coef = -0.99±0.35, p = 0.004). The scatterplot of the effect sizes against the quality of reporting showed that BIC decreased from approximately 65% for studies with low quality to 50% for studies with high quality (Fig 2B). No significant differences were observed for study funding (p = 0.06).

**Fig 4:**
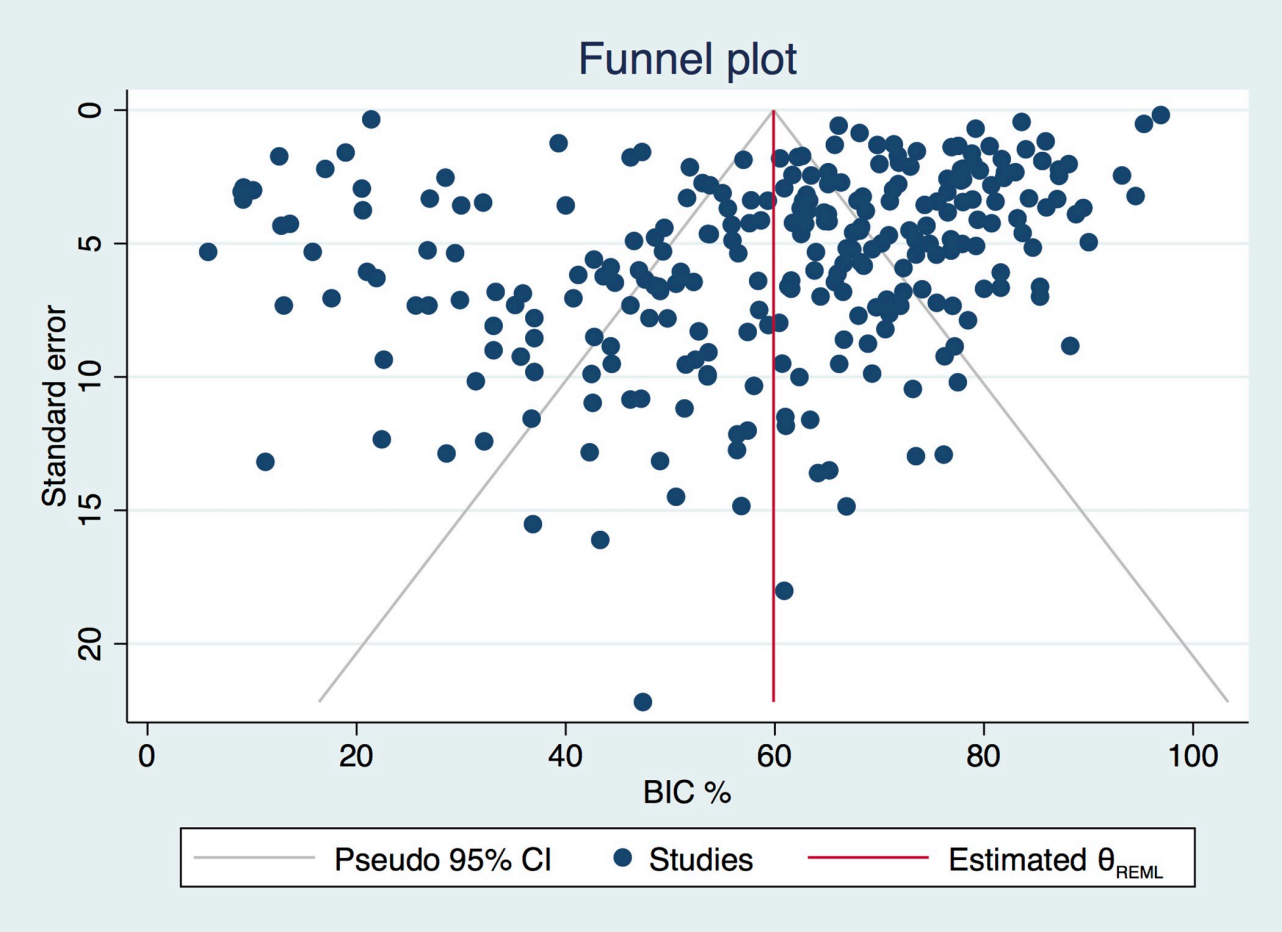
Funnel plot.

Heterogeneity was high for all random effect models (I^2^ > 90%). Evidence of publication bias was observed in the funnel plot (Fig 4) and the Egger test was statistically significant (p<0.001).

### Secondary outcomes

Table 3 presents first BIC and crestal bone level according to implant site. Nine studies reported combined buccal and lingual sites first BIC averaging 1.24mm (95%CI: 0.83–1.66). Four studies reported buccal and lingual sites separately first BIC averaging 1.5mm. Four studies reported crestal bone level separately for buccal and lingual sites mean bone level approximating 1.5mm. No studies reported crestal bone level combining buccal and lingual sites.

**Table 3:** Crestal bone level and first BIC according to implant site (in mm).

	Site	n studies	Mean (95% CI)	I^2^	p-value
First bone-implant contact	Buccal	4	1.65 (1.23–2.07)	91.37%	<0.001
	Lingual	4	1.55 (1.11–2.00)	93.56%	<0.001
	Buccal+Lingual	9	1.24 (0.83–1.66)	98.19%	<0.001
Crestal bone level	Buccal	4	1.34 (0.81–1.86)	100.00%	<0.001
	Lingual	4	1.36 (0.78–1.94)	97.99%	<0.001
	Buccal+Lingual	NA	NA		

BIC: Bone-implant contact; CI: Confidence interval; I^2^: Heterogeneity index; NA: Not available.

## Discussion

In summary, the present systematic review included 55 studies evaluating osseointegration and crestal bone remodeling using a minipig intraoral dental implant model. Most studies evaluated novel dental implant surfaces. Great variability in minipig strain and age, sample size, healing time, and surgical approach was observed. Approximately 90% of studies reported undecalcified histology and incandescent light microscopy histometrics. The quality of reporting assessment identified that most studies did not sufficiently report several methodological items, including animal characteristics and husbandry, sample size calculation, examiner calibration. masking/blinding, and statistical analysis. Studies typically extracted the mandibular premolar and first molar teeth and allowed 12 wks post-extraction healing. Three narrow, 8–10 mm implants were placed in contralateral jaw quadrants and allowed to osseointegrate submerged for 8 wks. The overall mean BIC was approximately 60% for the minipig intraoral dental implant model; BIC increased steadily during the first 5–6 wks and remained stable onwards. Between-study heterogeneity could be explained by healing time post-extraction and after implant placement, and implant surface. Few studies evaluated bone remodeling around the implant platform; the mean first BIC distance was approximately 1.2mm and crestal bone level was 1.5mm.

Few studies have evaluated dental implant osseointegration in humans [70–77]. For instance, Lang et al. (2011) compared osseointegration of two sandblasted acid-etched surface mini-implants (SLA and SLActive, Straumann^®^, Basel, Switzerland) in the posterior mandible [71]. BIC increased from 12.2–14.8% wk 2, to 32.4–48.3% wk 4, to 62% wk 6. Cecchinato et al. (2012) evaluated osseointegration of a fluoride-treated nanostructured mini-implant (Osseospeed^®^, Astra, Charlotte, NC USA) in individuals with and without history of periodontitis [72]. Overall BIC averaged 58.4±13.0% following 12 wks of osseointegration. Still others observed a mean BIC ranging from 45% to 75% following 12 wks of osseointegration depending on site characteristics and surgical/loading protocols [73,74,76]. Collectively, these estimates of osseointegration are comparable with mean BICs observed in minipigs ranging from 40.9% to 69.1% depending on observation interval. Nevertheless, whereas large animal models may provide estimates of osseointegration comparable with that in humans, it is prudent to caution that bone formation/remodeling [78] and osseointegration [79] appears faster than in humans.

From a regulatory standpoint, several agencies, including the United States Food and Drug Administration, follow the technical specifications related to preclinical evaluation of dental implant systems outlined by the International Organization for Standardization [80]. The specifications indicate that predicate implant devices intended for human clinical use should be tested in intraoral sites with opposing teeth. The animals should have a non-herbivorous pattern of masticatory jaw movements and allow for long-term oral hygiene to be maintained. Although domestic pigs have been used to test dental implants [81–84], their increased size and weight at an early age leads to challenges in husbandry and handling [85]. Nonhuman primate and canine models also fulfill these requirements, however their use has been logistically challenging opposed by public opinion [2,86,87]. In comparison to canines, minipigs require more specialized facilities and veterinary care; animal availability and cost might be an issue depending on age/sex and number of authorized vendors.

For decades the canine model has been the preferred platform in implant dentistry due to its availability, handling, anatomic and biologic similarities. Several studies have observed comparable osseointegration rates for the canine and minipig intraoral implant models [88]. A meta-analysis comparing titanium and zirconia implants reported an overall BIC of 60.4% (95%CI: 52.8–67.9%) for titanium implants using a wide range of healing intervals [89]. Abrahamsson et al. (2004) observed a BIC approximating 60% at 12 wks evaluating sandblasted and acid-etched implants [90]. Cochran et al. (1998) reported a mean BIC of 68% for SLA and 78% for titanium plasma sprayed implants at 12 months indicating stable long-term osseointegration [29]. Our laboratory has demonstrated BICs ranging between 63% and 78% for anodized implants at 8 wks in a series of studies evaluating surgical techniques, implant materials, surface characteristics, and restorative approaches [91–93].

The quality assessment of reporting in this review show a need for more stringent reporting that readers can evaluate the quality of the studies and researchers replicate methodologies. Only one study was judged to provide a complete description of the methods and results; most studies exhibited multiple omissions. Future reports using the minipig intraoral dental implant model should follow the ARRIVE guidelines [10]. Special attention should be given to sample size calculation, randomization, and examiner masking/blinding to minimize the number of underpowered studies and risk of bias. We did not formally apply established risk of bias tools for animal research such as SYRCLE [94] due to the difficulty to adapt its use to large animal studies and large number of studies that did not report methodology appropriately. Nevertheless, an exploratory analysis showed an inverse relationship between quality of reporting and osseointegration, which may indicate some inflation in the estimates.

This systematic review underscores the safety and efficacy of the surgical procedures and implant technologies tested by most studies using the minipig intraoral dental implant model as measured by clinically acceptable levels of osseointegration, crestal remodeling and short-term survival rates. In perspective, the cumulative implant failure rate in humans for commercially available implants with moderately rough surfaces reviewed herein has been estimated to be approximately 4% after 10 or more years in function [95]. This provides indirect evidence that the osseointegration level observed within 3–4 months following implant placement in minipigs could translate into meaningful long-term clinical outcomes for patients barring technical and biological complications.

The experimental design complexity, including multiple experimental groups and healing times, observed in this review underscores the tension between a desire to reduce the number of animals used in research, one of the pillars of the 3Rs by Russel and Burch [96], while collecting as much data as possible within a single experiment. However well intentioned, this approach is clearly generating a high level of data heterogeneity, which contributes to unreliable results and potentially to reporting bias. The use of simplified study designs such as the split-mouth design with multiple observations per experimental group/animal (duplicates, triplicates) would likely yield most robust results.

## Conclusions

Despite reported great variability observed, preferred characteristics for the minipig intraoral dental implant model have emerged, including observation intervals, implant placement approaches, number and size of implants, and outcomes assessment. Osseointegration estimates were comparable to other large animal models and human studies indicating that the minipig model can provide meaningful information for clinical applications.

## Supporting information

S1 Checklist PRISMA 2020 checklist. (PDF)

S1 Table Items evaluated in the quality assessment–adapted from ARRIVE checklist. (DOCX)

S2 Table Excluded full-texts and reasons (n = 70). (DOCX)

S3 Table Description of the 55 studies included in the systematic review. (DOCX)

S4 Table Quality assessment of included studies (based on ARRIVE checklist). (DOCX)
