# Supplementary material for: The minipig intraoral dental implant model: A systematic review and meta-analysis
Source: PLoS One. 2022 Feb 28;17(2):e0264475. doi: 10.1371/journal.pone.0264475 (PMC8884544; doi:10.1371/journal.pone.0264475)
Supplement: S1 Table — (DOCX) [file pone.0264475.s002.docx]

Supplemental Table 1. Items evaluated in the quality assessment – adapted from ARRIVE checklist

| METHODS | Item | Recommendation |
| --- | --- | --- |
| Study design | 6 | For each experiment, give brief details of the study design, including:  a. The number of experimental and control groups.  b. Any steps taken to minimise the effects of subjective bias when allocating animals to treatment (e.g.,  randomisation procedure) and when assessing results (e.g., if done, describe who was blinded and when).  c. The experimental unit (e.g. a single animal, group, or cage of animals).  A time-line diagram or flow chart can be useful to illustrate how complex study designs were carried out. |
| Experimental procedures | 7 | For each experiment and each experimental group, including controls, provide precise details of all  procedures carried out. For example:  a. How (e.g., drug formulation and dose, site and route of administration, anaesthesia and analgesia  used [including monitoring], surgical procedure (implant surgical protocol), method of euthanasia). Provide details of any specialist  equipment used, including supplier(s). |
| Experimental animals | 8 | a. Provide details of the animals used, including species, strain, sex, developmental stage (e.g., mean or  median age plus age range), and weight (e.g., mean or median weight plus weight range). |
| Housing and husbandry | 9 | Provide details of:  a. Housing (e.g., type of facility, e.g., specific pathogen free (SPF); type of cage or housing; bedding  material; number of cage companions; tank shape and material etc. for fish).  b. Husbandry conditions (e.g., breeding programme, light/dark cycle, temperature, quality of water etc.  for fish, type of food, access to food and water, environmental enrichment).  c. Welfare-related assessments and interventions that were carried out before, during, or after the  experiment. |
| Sample size | 10 | a. Specify the total number of animals used in each experiment and the number of animals in each  experimental group.  b. Explain how the number of animals was decided. Provide details of any sample size calculation used. |
| Allocating animals to  experimental groups | 11 | a. Give full details of how animals were allocated to experimental groups, including randomisation or  matching if done. |
| RESULTS |  |  |
| Numbers analysed | 15 | a. Report the number of animals in each group included in each analysis. Report absolute numbers (e.g.  10/20, not 50%a). |
| Adverse events |  |  |
|  | 17 | 1. Give details of all important adverse events in each experimental group. |
