# Supplementary material for: The minipig intraoral dental implant model: A systematic review and meta-analysis
Source: PLoS One. 2022 Feb 28;17(2):e0264475. doi: 10.1371/journal.pone.0264475 (PMC8884544; doi:10.1371/journal.pone.0264475)
Supplement: S2 Table — (DOCX) [file pone.0264475.s003.docx]

Supplemental Table 2. Excluded full-texts and reasons (n=70)

| He et al. 2020 | BIC was not reported |
| --- | --- |
| Jin H et al. 2020 | Implants placed in defects/augmented sites |
| Lyu et al. 2020 | Implants placed in defects/augmented sites |
| Yu et al. 2020 | Implants placed in extraoral sites (calvaria) |
| Beltran-Salinas et al. 2019 | Only abstract available |
| Caballe-Serrano et al. 2019 | BIC was not reported (same sample as Chappuis et al. 2016) |
| El-Chaar et al. 2019 | Implants placed in defects/augmented sites |
| Fernandez-Yague et al. 2019 | BIC standard deviation reported only in graph format |
| Grobecker-Karl et al. 2019 | Animals euthanized immediately after implant placement |
| Bissinger et al. 2018 | BIC standard deviation not reported |
| Chappuis et al. 2018 | BIC mean/standard deviation reported only in graph format |
| Coelho et al. 2018 | BIC mean/standard deviation reported only in graph format |
| Kubasiewicz-Ross et al. 2018 | BIC standard deviation was not reported |
| Bissinger et al. 2017 | BIC standard deviation was not reported |
| Chien et al. 2017 | Implants placed in defects/augmented sites |
| Grobe et al. 2017 | BIC was not reported |
| Zhou et al. 2017 | BIC was not reported (same sample as Zechner et al. 2003a) |
| Chappuis et al. 2016 | BIC mean/standard deviation reported only in graph format |
| Gao et al. 2016 | BIC was not reported |
| Kalemaj et al. 2016 | Implants placed in extraoral sites (tibia) |
| Linares et al. 2016 | Duplicate data - Linares et al. 2013 |
| Wen et al. 2016 | No histology |
| Korn et al. 2015 | Only abstract available |
| Liñares et al. 2015 | BIC was not reported |
| Schierano et al. 2015 | Implants placed in extraoral sites (tibia) |
| Stricker et al. 2015 | Implants placed in defects/augmented sites |
| Anchieta et al. 2014 | BIC was not reported |
| Elian et al. 2014a | No histology |
| Elian et al. 2014b | No histology |
| Gil et al. 2014 | BIC mean/standard deviation reported only in graph format |
| Hasturk et al. 2014 | BIC mean/standard deviation reported only in graph format |
| Poort et al. 2014 | No histology |
| Saulacic et al. 2014 | Zirconia implants were used |
| Durual et al. 2013 | BIC mean/standard deviation reported only in graph format |
| Heitz-Mayfield et al. 2013 | BIC was not reported |
| Kang et al. 2013 | Implants placed in defects/augmented sites |
| Schultze-Mosgau et al. 2013 | Only abstract available |
| Schulz et al. 2013 | Only abstract available |
| Sprecher et al. 2013 | BIC was not reported (same sample as Gahlert et al. 2012, different outcome reported) |
| Bormann et al. 2012 | No histology |
| Farronato et al. 2012 | BIC was not reported |
| Hunziker et al. 2012 | BIC was not reported |
| Mueller et al. 2012a | BIC was not reported |
| Mueller et al. 2012b | BIC was not reported |
| Stadlinger et al. 2012b | No histology |
| Zambon et al. 2012 | Implants placed in defects/augmented sites |
| Aparicio et al. 2011 | BIC mean/standard deviation reported only in graph format |
| Grüner et al. 2011 | BIC was not reported |
| Mueller et al. 2011 | BIC was not reported |
| Santos Corpas et al. 2011 | BIC was not reported |
| Koch et al. 2010 | Only abstract available |
| Mueller et al. 2010 | BIC was not reported |
| Carmagnola et al. 2009 | BIC standard deviation not reported |
| Li et al. 2008 | BIC was not reported |
| Gahlert et al. 2007 | BIC was not reported |
| Liu et al. 2007 | BIC mean/standard deviation reported only in graph format |
| Neugebauer et al. 2006 | BIC was not reported |
| Nkenke et al. 2005b | BIC was not reported |
| Meyer et al. 2004 | BIC was not reported |
| Chang et al. 2003 | No histology |
| Ko et al. 2003 | BIC was not reported |
| Meyer et al. 2003 | BIC was not reported |
| Zechner et al. 2003b | Same sample as Zechner et al. 2003a (different comparison) |
| Li et al. 2002 | No histology |
| Buser et al. 1998 | No histology |
| Smith et al. 1995 | BIC standard deviation not reported |
| Schliephake et al. 1993 | BIC was not reported |
| Hale et al. 1991 | BIC standard deviation not reported |
| Hickey et al. 1991 | No histology |
| Karagianes et al. 1976 | BIC was not reported |

BIC: bone-implant contact.

References

1. He X, Reichl FX, Milz S, Michalke B, Wu X, Sprecher CM, et al. Titanium and zirconium release from titanium- and zirconia implants in mini pig maxillae and their toxicity in vitro. Dent Mater. 2020;36(3):402-12.

2. Jin HY, Teng MH, Wang D, Li X, Liang JY, Wang WX, et al. Modified Disk-Up Sinus Reamer for Sinus Floor Elevation and Simultaneous Implant Placement: An Animal Study with Miniature Pigs. J Invest Surg. 2020;33(6):520-9.

3. Lyu HZ, Lee JH. The efficacy of rhBMP-2 loaded hydrogel composite on bone formation around dental implants in mandible bone defects of minipigs. Biomater Res. 2020;24:5.

4. Yu W, Wang X, Guo Y, Yang S, Zhou Z, Sun X, et al. The osteogenesis performance of titanium modified via plasma-enhanced chemical vapor deposition: in vitro and in vivo studies. Biomed Mater. 2020;15(5):055012.

5. Beltran-Salinas B, López-Sosa L, Contreras-Hernandez G, Guzmán-García M, Hernandez-Rodriguez MAL. Evaluation of titanium dental implants with a bioactive surface treatment: histological comparative study using a minipig model. International Journal of Oral and Maxillofacial Surgery. 2019;48:153.

6. Caballé‐Serrano J, Chappuis V, Monje A, Buser D, Bosshardt DD. Soft tissue response to dental implant closure caps made of either polyetheretherketone (PEEK) or titanium. Clinical Oral Implants Research. 2019;30(8):808-16.

7. El Chaar E, Zhang L, Zhou Y, Sandgren R, Fricain JC, Dard M, et al. Osseointegration of Superhydrophilic Implants Placed in Defect Grafted Bones. Int J Oral Maxillofac Implants. 2019;34(2):443-50.

8. Fernandez-Yague M, Antonanzas RP, Roa JJ, Biggs M, Gil FJ, Pegueroles M. Enhanced osteoconductivity on electrically charged titanium implants treated by physicochemical surface modifications methods. Nanomedicine. 2019;18:1-10.

9. Grobecker‐Karl T, Palarie V, Schneider S, Karl M. Does intraoperative bone density testing correlate with parameters of primary implant stability? A pilot study in minipigs. Clinical and Experimental Dental Research. 2019;5(6):594-600.

10. Bissinger O, Gotz C, Jeschke A, Haller B, Wolff KD, Kaiser P, et al. Comparison of contact radiographed and stained histological sections for osseointegration analysis of dental implants: an in vivo study. Oral Surg Oral Med Oral Pathol Oral Radiol. 2018;125(1):20-6.

11. Chappuis V, Maestre L, Burki A, Barre S, Buser D, Zysset P, et al. Osseointegration of ultrafine-grained titanium with a hydrophilic nano-patterned surface: an in vivo examination in miniature pigs. Biomater Sci. 2018;6(9):2448-59.

12. Coelho PG, Pippenger B, Tovar N, Koopmans SJ, Plana NM, Graves DT, et al. Effect of Obesity or Metabolic Syndrome and Diabetes on Osseointegration of Dental Implants in a Miniature Swine Model: A Pilot Study. J Oral Maxillofac Surg. 2018;76(8):1677-87.

13. Kubasiewicz-Ross P, Hadzik J, Dominiak M. Osseointegration of zirconia implants with 3 varying surface textures and a titanium implant: A histological and micro-CT study. Adv Clin Exp Med. 2018;27(9):1173-9.

14. Bissinger O, Probst FA, Wolff KD, Jeschke A, Weitz J, Deppe H, et al. Comparative 3D micro-CT and 2D histomorphometry analysis of dental implant osseointegration in the maxilla of minipigs. J Clin Periodontol. 2017;44(4):418-27.

15. Chien SK HS, Lin CS, Kuo TF, Wang DJ, Yang JC et al. . Influence of Thread Design on Dental Implant Osseointegration Assayed Using the Lan-Yu Mini-Pig Model. Journal of Medical and Biological Engineering. 2017;37(5):627-38.

16. Grobe A, Semmusch J, Schollchen M, Hanken H, Hahn M, Eichhorn W, et al. Accuracy of Bone Measurements in the Vicinity of Titanium Implants in CBCT Data Sets: A Comparison of Radiological and Histological Findings in Minipigs. Biomed Res Int. 2017;2017:3848207.

17. Zhou W, Kuderer S, Liu Z, Ulm C, Rausch-Fan X, Tangl S. Peri-implant bone remodeling at the interface of three different implant types: a histomorphometric study in mini-pigs. Clin Oral Implants Res. 2017;28(11):1443-9.

18. Chappuis V, Cavusoglu Y, Gruber R, Kuchler U, Buser D, Bosshardt DD. Osseointegration of Zirconia in the Presence of Multinucleated Giant Cells. Clin Implant Dent Relat Res. 2016;18(4):686-98.

19. Gao ZH, Hu L, Liu GL, Wei FL, Liu Y, Liu ZH, et al. Bio-Root and Implant-Based Restoration as a Tooth Replacement Alternative. J Dent Res. 2016;95(6):642-9.

20. Kalemaj Z, Scarano A, Valbonetti L, Rapone B, Grassi FR. Bone Response to Four Dental Implants with Different Surface Topographies: A Histologic and Histometric Study in Minipigs. Int J Periodontics Restorative Dent. 2016;36(5):745-54.

21. Linares A, Grize L, Munoz F, Pippenger BE, Dard M, Domken O, et al. Histological assessment of hard and soft tissues surrounding a novel ceramic implant: a pilot study in the minipig. J Clin Periodontol. 2016;43(6):538-46.

22. Wen B, Chen J, Dard M, Cai Z. The Performance of Titanium-Zirconium Implants in the Elderly: A Biomechanical Comparative Study in the Minipig. Clin Implant Dent Relat Res. 2016;18(6):1200-9.

23. Korn P, Elschner, C., Schulz, M.C., Range, U., Mai, R., Scheler, U. Titanium coated peek implants as basis for multimodal imaging in implant research

. International Journal of Oral and Maxillofacial Surgery. 2015;44.

24. Linares A, Munoz F, Permuy M, Dard M, Blanco J. Soft tissue histomorphology at implants with a transmucosal modified surface. A study in minipigs. Clin Oral Implants Res. 2015;26(9):996-1005.

25. Schierano G, Mussano F, Faga MG, Menicucci G, Manzella C, Sabione C, et al. An alumina toughened zirconia composite for dental implant application: in vivo animal results. Biomed Res Int. 2015;2015:157360.

26. Stricker A, Fleiner J, Stubinger S, Schmelzeisen R, Dard M, Bosshardt DD. Bone loss after ridge expansion with or without reflection of the periosteum. Clin Oral Implants Res. 2015;26(5):529-36.

27. Anchieta RB, Baldassarri M, Guastaldi F, Tovar N, Janal MN, Gottlow J, et al. Mechanical property assessment of bone healing around a titanium-zirconium alloy dental implant. Clin Implant Dent Relat Res. 2014;16(6):913-9.

28. Elian N, Bloom M, Dard M, Cho SC, Trushkowsky RD, Tarnow D. Radiological and micro-computed tomography analysis of the bone at dental implants inserted 2, 3 and 4 mm apart in a minipig model with platform switching incorporated. Clin Oral Implants Res. 2014;25(2):e22-9.

29. Elian N, Bloom M, Trushkowsky RD, Dard MM, Tarnow D. Effect of 3- and 4-mm interimplant distances on the height of interimplant bone crest: a histomorphometric evaluation measured on bone level dental implants in minipig. Implant Dent. 2014;23(5):522-8.

30. Gil FJ, Manzanares N, Badet A, Aparicio C, Ginebra MP. Biomimetic treatment on dental implants for short-term bone regeneration. Clin Oral Investig. 2014;18(1):59-66.

31. Hasturk H, Kantarci A, Ghattas M, Dangaria SJ, Abdallah R, Morgan EF, et al. The use of light/chemically hardened polymethylmethacrylate, polyhydroxylethylmethacrylate, and calcium hydroxide graft material in combination with polyanhydride around implants and extraction sockets in minipigs: Part II: histologic and micro-CT evaluations. J Periodontol. 2014;85(9):1230-9.

32. Poort LJ, Bittermann GK, Bockmann RA, Hoebers FJ, Houben R, Postma AA, et al. Does a change in bone mineral density occur in the mandible of Gottingen minipigs after irradiation in correlation with radiation dose and implant surgery? J Oral Maxillofac Surg. 2014;72(11):2149-56.

33. Saulacic N, Erdosi R, Bosshardt DD, Gruber R, Buser D. Acid and alkaline etching of sandblasted zirconia implants: a histomorphometric study in miniature pigs. Clin Implant Dent Relat Res. 2014;16(3):313-22.

34. Durual S, Rieder P, Garavaglia G, Filieri A, Cattani-Lorente M, Scherrer SS, et al. TiNOx coatings on roughened titanium and CoCr alloy accelerate early osseointegration of dental implants in minipigs. Bone. 2013;52(1):230-7.

35. Heitz-Mayfield LJ, Darby I, Heitz F, Chen S. Preservation of crestal bone by implant design. A comparative study in minipigs. Clin Oral Implants Res. 2013;24(3):243-9.

36. Kang EJ, Kim SK, Eom TG, Choi KO, Lee TH. Evaluation of the osteogenic activity of the BMP-2 mimetic peptide, PEP7, in vitro and in vivo. Int J Oral Maxillofac Implants. 2013;28(3):749-56.

37. Schultze-Mosgau S. Concepts of flapless surgery for implantation. International Journal of Oral and Maxillofacial Surgery. 2013;42(10):1267.

38. Schulz M, Korn P, Stadlinnger B, Range U, Hintze V. Coating with sulphated hyaluronan enhances osseointegration of dental implants. International Journal of Oral and Maxillofacial Surgery. 2013;42(10):1268.

39. Sprecher CM, Gahlert M, Rohling S, Kniha H, Gueorguiev B, Milz S. Comparison of imaging methods used for dental implant osseous integration assessment. J Mater Sci Mater Med. 2013;24(9):2195-200.

40. Bormann KH, Gellrich NC, Kniha H, Dard M, Wieland M, Gahlert M. Biomechanical evaluation of a microstructured zirconia implant by a removal torque comparison with a standard Ti-SLA implant. Clin Oral Implants Res. 2012;23(10):1210-6.

41. Farronato D, Santoro G, Canullo L, Botticelli D, Maiorana C, Lang NP. Establishment of the epithelial attachment and connective tissue adaptation to implants installed under the concept of "platform switching": a histologic study in minipigs. Clin Oral Implants Res. 2012;23(1):90-4.

42. Hunziker EB, Enggist L, Kuffer A, Buser D, Liu Y. Osseointegration: the slow delivery of BMP-2 enhances osteoinductivity. Bone. 2012;51(1):98-106.

43. Mueller CK, Thorwarth M, Schultze-Mosgau S. Analysis of inflammatory periimplant lesions during a 12-week period of undisturbed plaque accumulation--a comparison between flapless and flap surgery in the mini-pig. Clin Oral Investig. 2012;16(2):379-85.

44. Mueller CK, Thorwarth M, Chen J, Schultze-Mosgau S. A laboratory study comparing the effect of ridge exposure using tissue punch versus mucoperiosteal flap on the formation of the implant-epithelial junction. Oral surgery, oral medicine, oral pathology and oral radiology. 2012;114(5):S41-S5.

45. Stadlinger B, Ferguson SJ, Eckelt U, Mai R, Lode AT, Loukota R, et al. Biomechanical evaluation of a titanium implant surface conditioned by a hydroxide ion solution. Br J Oral Maxillofac Surg. 2012;50(1):74-9.

46. Zambon R, Mardas N, Horvath A, Petrie A, Dard M, Donos N. The effect of loading in regenerated bone in dehiscence defects following a combined approach of bone grafting and GBR. Clin Oral Implants Res. 2012;23(5):591-601.

47. Aparicio C, Padros A, Gil FJ. In vivo evaluation of micro-rough and bioactive titanium dental implants using histometry and pull-out tests. J Mech Behav Biomed Mater. 2011;4(8):1672-82.

48. Gruner D, Faldt J, Jansson K, Shen Z. Argon ion beam polishing: a preparation technique for evaluating the interface of osseointegrated implants with high resolution. Int J Oral Maxillofac Implants. 2011;26(3):547-52.

49. Mueller CK, Thorwarth M, Schultze-Mosgau S. Histomorphometric and whole-genome expression analysis of peri-implant soft tissue healing: a comparison of flapless and open surgery. Int J Oral Maxillofac Implants. 2011;26(4):760-7.

50. dos Santos Corpas L, Jacobs R, Quirynen M, Huang Y, Naert I, Duyck J. Peri‐implant bone tissue assessment by comparing the outcome of intra‐oral radiograph and cone beam computed tomography analyses to the histological standard. Clinical oral implants research. 2011;22(5):492-9.

51. Koch C. KA, Probst F A, Bissinger O, Weitz J, Bauer F, Plank C. Acceleration of osseointegration by nanoparticle loaded dental implants: A study in mini pigs. Human Gene Therapy. 2010;21.

52. Mueller CK, Thorwarth M, Schultze-Mosgau S. Influence of insertion protocol and implant shoulder design on inflammatory infiltration and gene expression in peri-implant soft tissue during nonsubmerged dental implant healing. Oral Surg Oral Med Oral Pathol Oral Radiol Endod. 2010;109(5):e11-9.

53. Carmagnola D, Abati S, Addis A, Ferrieri G, Chiapasco M, Romeo E, et al. Time sequence of bone healing around two implant systems in minipigs: preliminary histologic results. Int J Periodontics Restorative Dent. 2009;29(5):549-55.

54. Li Y, Lee IS, Cui FZ, Choi SH. The biocompatibility of nanostructured calcium phosphate coated on micro-arc oxidized titanium. Biomaterials. 2008;29(13):2025-32.

55. Gahlert M, Gudehus T, Eichhorn S, Steinhauser E, Kniha H, Erhardt W. Biomechanical and histomorphometric comparison between zirconia implants with varying surface textures and a titanium implant in the maxilla of miniature pigs. Clin Oral Implants Res. 2007;18(5):662-8.

56. Liu Y, Enggist L, Kuffer AF, Buser D, Hunziker EB. The influence of BMP-2 and its mode of delivery on the osteoconductivity of implant surfaces during the early phase of osseointegration. Biomaterials. 2007;28(16):2677-86.

57. Neugebauer J, Traini T, Thams U, Piattelli A, Zoller JE. Peri-implant bone organization under immediate loading state. Circularly polarized light analyses: a minipig study. J Periodontol. 2006;77(2):152-60.

58. Nkenke E, Lehner B, Fenner M, Roman FS, Thams U, Neukam FW, et al. Immediate versus delayed loading of dental implants in the maxillae of minipigs: follow-up of implant stability and implant failures. Int J Oral Maxillofac Implants. 2005;20(1):39-47.

59. Meyer U, Joos U, Mythili J, Stamm T, Hohoff A, Fillies T, et al. Ultrastructural characterization of the implant/bone interface of immediately loaded dental implants. Biomaterials. 2004;25(10):1959-67.

60. Chang MC, Ko CC, Liu CC, Douglas WH, DeLong R, Seong WJ, et al. Elasticity of alveolar bone near dental implant-bone interfaces after one month's healing. J Biomech. 2003;36(8):1209-14.

61. Ko CC, Douglas WH, DeLong R, Rohrer MD, Swift JQ, Hodges JS, et al. Effects of implant healing time on crestal bone loss of a controlled-load dental implant. J Dent Res. 2003;82(8):585-91.

62. Meyer U, Wiesmann H-P, Fillies T, Joos U. Early tissue reaction at the interface of immediately loaded dental implants. International Journal of Oral & Maxillofacial Implants. 2003;18(4).

63. Zechner W, Tangl S, Furst G, Tepper G, Thams U, Mailath G, et al. Osseous healing characteristics of three different implant types. Clin Oral Implants Res. 2003;14(2):150-7.

64. Li D, Ferguson SJ, Beutler T, Cochran DL, Sittig C, Hirt HP, et al. Biomechanical comparison of the sandblasted and acid-etched and the machined and acid-etched titanium surface for dental implants. J Biomed Mater Res. 2002;60(2):325-32.

65. Buser D, Nydegger T, Hirt HP, Cochran DL, Nolte L-P. Removal torque values of titanium implants in the maxilla of miniature pigs. International Journal of Oral & Maxillofacial Implants. 1998;13(5).

66. Smith RA. The effect on TGF-beta 1 on osseointegration. J Calif Dent Assoc. 1995;23(12):49-53.

67. Schliephake H, Reiss G, Urban R, Neukam F, Guckel S. Metal release from titanium fixtures during placement in the mandible: an experimental study. International Journal of Oral & Maxillofacial Implants. 1993;8(5).

68. Hale TM, Boretsky BB, Scheidt MJ, McQuade MJ, Strong SL, Van Dyke TE. Evaluation of titanium dental implant osseointegration in posterior edentulous areas of micro swine. J Oral Implantol. 1991;17(2):118-24.

69. Hickey JS, O'Neal RB, Scheidt MJ, Strong SL, Turgeon D, Van Dyke TE. Microbiologic characterization of ligature-induced peri-implantitis in the microswine model. J Periodontol. 1991;62(9):548-53.

70. Karagianes MT, Westerman RE, Rasmussen JJ, Lodmell AM. Development and evaluation of porous dental implants in miniature swine. J Dent Res. 1976;55(1):85-93.
