# Supplementary material for: The minipig intraoral dental implant model: A systematic review and meta-analysis
Source: PLoS One. 2022 Feb 28;17(2):e0264475. doi: 10.1371/journal.pone.0264475 (PMC8884544; doi:10.1371/journal.pone.0264475)
Supplement: S3 Table — (DOCX) [file pone.0264475.s004.docx]

Supplemental Table 3. Description of the 55 studies included in the systematic review

|  | **Surgical extraction**  **Arch**  **Teeth extracted**  **Healing period** | **Implant placement**  **# implants**  **Implant size**  **Surface characteristics**  **Staging**  **Healing period** | **Histology**  **Histological processing**  **Histological analysis**  **Direction**  **Staining**  **Section thickness** | **Main findings**  **Bone-to-implant contact %**  **mean/med (SD/SE)**  **according to healing time** |
| --- | --- | --- | --- | --- |
| Hoornaert et al. (2020)  6 Göttingen minipigs  all females  18 months old | maxilla and mandible  all pre-molars and 1^st^ molars  12 weeks | 60 implants  Ø3.4x8mm  Anthogyr^®^; Biotech Dental Kontact^®^  delayed/submerged  4 and 12 weeks | mineralized  back scattered SEM  unclear cut orientation  toluidine blue + basic fuchsin  10 μm | \|  \| Implant surface \| \| \| --- \| --- \| --- \| \| weeks \| Anthogyr^®^ \| Biotech Dental^®^ \| \| 4 max \| 21(10.5) \| 35.9(11.9) \| \| 4 man \| 12.6(3) \| 35.7(16) \| \| 12 max \| 20.6(6.5) \| 21.9(10.9) \| \| 12 man \| 32.2(21.5) \| 47(10.4) \| |
| Kämmerer et al. (2020)  9 unspecified minipigs  all females  60-96 months old | mandible  pre-molars  12 weeks | 36 implants  Ø4.2x6mm  SA (sandblasted and acid-etched) and CaP coated (BONIT^®^)  delayed/unclear healing type  2, 4, and 8 weeks | mineralized  light microscopy  unclear cut orientation  toluidine blue  30-40 μm | \|  \| Implant surface \| \| \| --- \| --- \| --- \| \| weeks \| SA \| CaP coated \| \| 2 \| 21.4(0.6) \| 5.8(9.2) \| \| 4 \| 43.3(27.9) \| 32.1(6) \| \| 8 \| 12.8(7.5) \| 15.8(9.2) \| |
| Karl et al. (2020)  6 unspecified minipigs  unspecified sex  21.8 months old | mandible  all pre-molars  12 weeks | 24 implants  Ø3.75x7mm  TiUnite^®^  delayed/submerged  12, 18, and 24 weeks | mineralized  light microscopy  buccal-lingual  toluidine blue  70 μm | \|  \| Implant surface \| \| --- \| --- \| \| weeks \| TiUnite^®^ \| \| 12 \| 95.3(0.73) \| \| 18 \| 97.6(NA) \| \| 24 \| 96.91(0.26) \| |
| Thomé et al. (2020)  6 Göttingen minipigs  all females  20-24 months old | mandible  2^nd^ to 4^th^ pre-molars and 1^st^ molars  12 weeks | 36 implants  Ø4.3x10mm  Neodent^®^ Neoporos  delayed/submerged  8 weeks | mineralized  light microscopy  unclear cut orientation  toluidine blue + basic fuchsin  30-50 μm | \|  \| Implant surface \| \| --- \| --- \| \| weeks \| Neodent^®^ Neoporos \| \| 8 \| 80.7(6.9) \| |
| Romero-Ruiz et al. (2019)  12 unspecified minipigs  all females  72 months old | maxilla  all pre-molars and molars  24 weeks | 36 implants  Ø4.1x8mm  SLA^®^; SLActive^®^; ContactTi^®^  delayed/not submerged  2, 4, and 8 weeks | mineralized  back scattered SEM  unclear cut orientation  NA  0.008 μm | \|  \| Implant surface \| \| \| \| --- \| --- \| --- \| --- \| \| weeks \| SLA^®^ \| SLActive^®^ \| ContactTi^®^ \| \| 2 \| 53.56 \| 61.56 \| 62.35 \| \| (19.96) \| (13.39) \| (20.01) \| \| 4 \| 81.6 \| 85.36 \| 88.25 \| \| (13.31) \| (13.98) \| (17.67) \| \| 8 \| 77.99 \| 87.18 \| 90.02 \| \| (6.89) \| (4.93) \| (9.9) \| |
| Susin et al. (2019) a  24 Yucatan minipigs  females and castrated males  20-24 months old | mandible  3^rd^ and 4^th^ pre-molars and 1^st^ molar  16 weeks | 96 implants  Ø3.5x10mm  TiUnite^®^  delayed/not submerged  3, 6, and 13 weeks | mineralized  light microscopy  buccal-lingual  Stevenel’s Blue  35-55 μm | \|  \| Implant surface \| \| \| --- \| --- \| --- \| \| weeks \| TiUnite^®^ \| TiUltra^®^ \| \| 3 \| 59.33(9.60)* \| 63.12(10.68) \| \| 6 \| 65.07(11.03) \| 58.67(11.69) \| \| 13 \| 63.93(15.05) \| 57.73(9.56) \|   *buccal/lingual combined |
| Susin et al. (2019) b  12 Yucatan minipigs  females and castrated males  16-19 months old | mandible  3^rd^ and 4^th^ pre-molars and 1^st^ molars  15 weeks | 72 implants  Ø3.5x10mm  TiUnite^®^; TiUltra^®^  delayed/not submerged  6 and 13 weeks | mineralized  light microscopy  buccal-lingual  Sanderson's RBS and acid fuchsin  50-100 μm | \|  \| \| Abutment surface \| \| \| --- \| --- \| --- \| --- \| \| weeks \| TiUnite^®^ \| \| TiUltra^®^ \| \| 6 \| 60.90(7.17)* \| \| 53.78(11.39) \| \| 13 \| 66.33(6.64) \| \| 65.73(3.19) \|   *buccal/lingual combined |
| Herrero-Climent et al. (2018)  12 unspecified minipigs  all females  72 months old | maxilla  pre-molars and molars  16 weeks | 48 implants  Ø4x8mm  Shotblasting; ContactTi^®^  Delayed/3 months submerged + 6 months not submerged  2, 4, and 8 weeks | mineralized  back scattered SEM  buccal-lingual  NA  0.008 μm | \|  \| Implant surface \| \| \| --- \| --- \| --- \| \| weeks \| Shotbalsting \| ContactTi^®^ \| \| 2 \| 39.32(2.48) \| 49.02(26.30) \| \| 4 \| 46.53(9.81) \| 83.20(8.12) \| \| 8 \| 46.20(3.54) \| 85.58(3.81) \| |
| Hou et al. (2018)  6 Sus barbatus sumatranus minipigs  unspecified sex  unspecified age | mandible  no extraction  NA (implants placed in edentulous areas between teeth) | 12 implants  Ø3.5x8mm  Machined; MST-Ti (micro-arc oxidation surface-treated)  NA/unclear healing type  8 and 12 weeks | mineralized  light microscopy  unclear cut orientation  toluidine blue  thickness not reported | \|  \| Implant surface \| \| \| --- \| --- \| --- \| \| weeks \| machined \| MST-Ti \| \| 8 \| 70(3.5) \| 76.9(2.4) \| \| 12 \| 71.8(4.8) \| 88.1(3.5) \| |
| Mehl et al. (2018)  3 unspecified minipigs  2 males and 1 female  unspecified age | maxilla and mandible  all pre-molars and 1^st^ molars  12 weeks | 48 implants  Ø4.3x9mm  Promote^®^ (with or without UV functionalization)  delayed/3months submerged + 6 months not submerged  24 weeks | mineralized  light microscopy  buccal-lingual  toluidine blue  30-40 μm | \|  \| Maxilla \| \| Mandible \| \| \| --- \| --- \| --- \| --- \| --- \| \| weeks \| Not functionalized \| UV functionalized \| Not functionalized \| UV functionalized \| \| 24 \| 61.73  (7.32) \| 49.03  (11.74) \| 68.90  (15.16) \| 76.23  (15.98) \| |
| Ríos-Santos et al. (2018)  12 unspecified minipigs  all females  72 months old | maxilla  pre-molars and molars  24 weeks | 96 implants  Ø4.1x8mm  SLA^®^; SLActive^®^; Shotblasting^®^; ContactTi^®^; sandblasted and acid etched; sandblasted with alumina oxide + acid attack  delayed/submerged  2, 4, and 8 weeks | mineralized  back scattered SEM  unclear cut orientation  NA  thickness not reported | \| Surface \| 2 weeks \| 4 weeks \| 8 weeks \| \| --- \| --- \| --- \| --- \| \| SLA^®^  Straumann Tissue Level (STL) \| 53.56  (19.80) \| 81.6  (12.17) \| 80.73  (8.48) \| \| SLActive^®^  STL \| 61.56  (12.77) \| 85.36  (13.24) \| 87.17  (4.45) \| \| Shotblasting^®^  STL \| 50.55  (13.01) \| 53.65  (18.15) \| 70.70  (14.20) \| \| Shotblasting^®^  Essential cone design \| 49.73  (15.61) \| 51.48  (19.07) \| 47.3  (3.14) \| \| Shotblasting^®^  Prototype implant design \| 44.40  (19.02) \| 36.73  (23.12) \| 71.76  (3.41) \| \| ContactTi^®^  Essential cone design \| 36.86  (31.03) \| 83.2  (8.11) \| 85.58  (3.81) \| \| sandblasted and acid etched  Essential cone design \| 18.95  (3.18) \| 52.23  (12.88) \| 64.13  (27.20) \| \| sandblasted with alumina oxide + acid attack  Essential cone design \| 47.21  (21.63) \| 70.94  (15.26) \| 76.85  (10.53) \| |
| Kuo et al. (2017)  9 Lanyu small-ear pigs  unspecified sex  8-12 months old | maxilla and mandible  1^st^ to 3^rd^ pre-molars  12 weeks | 27 implants  Ø4.5x9mm  ComMed SLA  delayed/submerged  8 and 16 weeks | mineralized  back scattered SEM and light microscopy  unclear cut direction  Masson-Goldner Trichrome  thickness not reported | \|  \| Arch \| \| \| --- \| --- \| --- \| \| weeks \| maxilla \| mandible \| \| 8 \| 88.8(7.80) \| 93.2(4.90) \| \| 16 \| 89.50(8.20) \| 94.50(7.20) \|   *light microscopy reported (Table 4) |
| Brockmeyer et al. (2016)  30 Göttingen minipigs  all females  24 months old | maxilla and mandible  all pre-molars  12 weeks | 360 implants  Ø5x8mm  Inicell^®^  delayed/submerged  4, 12, 24, 48, and 96 weeks | mineralized  light microscopy  unclear cut direction  Smith-Karagianes  (methylene blue/alizarin red S)  25 μm | \|  \| Arch \| \| \| --- \| --- \| --- \| \| weeks \| maxilla \| mandible \| \| 4 \| 69.31(12.77) \| 68.12(11.06) \| \| 12 \| 70.90(11.49) \| 60.42(19.53) \| \| 24 \| 73.50(13.26) \| 70.57(20.11) \| \| 48 \| 66.52(16.68) \| 70.52(17.98) \| \| 96 \| 68.00(18.87) \| 64.37(17.11) \| \|  \|  \|  \| |
| Chiang et al. (2016)  12 Lanyu small-ear pigs  unspecified sex  12 months old | mandible  no extraction  NA (implants placed in edentulous areas between teeth) | 36 implants  Ø4.5x11mm  Machined; Hung Chun Bio-S SLA and SLAffinity  NA/submerged  3 and 6 weeks | mineralized  light microscopy  mesio-distal  toluidine blue  50 μm | \|  \| Implant surface \| \| \| \| --- \| --- \| --- \| --- \| \| weeks \| machined \| SLA \| SLAffinity \| \| 3 \| 65.14  (4.17) \| 66.87  (5.18) \| 72.87  (4.51) \| \| 6 \| 73.46  (4.87) \| 76.84  (4.84) \| 79.40  (4.11) \| |
| Cochran et al. (2016)  11 Göttingen minipigs  all females  30 months old | mandible  2^nd^, 3^rd^, and 4^th^ pre-molars and 1^st^ molars  12 weeks | 66 implants  Ø4.1x8mm  SLActive^®^  delayed/ 1 group submerged and 1 group not submerged  4 and 8 weeks | mineralized  light microscopy  buccal-lingual and mesio-distal  Paragon  30-50 μm | \|  \|  \| Implant design \| \| \| --- \| --- \| --- \| --- \| \| weeks \| loading \| Bone level \| Bone level tapered \| \| 4 \| no \| 68.20  (18.90) \| 64.80  (13.80) \| \| 8 \| 8 weeks \| 79.60  (7.50) \| 76.50  (10.50) \| |
| Eom et al. (2016)  10 unspecified minipigs  all males  24 months old | mandible  2^nd^, 3^rd^, and 4^th^ pre-molars and 1^st^ molars  32 weeks | 60 implants  Ø3.5x8.5mm  Osstem Implant^®^ - Resorbable blasted media  delayed/submerged  1, 3, and 5 weeks | mineralized  light microscopy  buccal-lingual  hematoxylin-eosin  thickness not reported | \|  \| Final osteotomy drill \| \| \| \| --- \| --- \| --- \| --- \| \| weeks \| 3.0mm \| 3.3mm \| 3.5mm \| \| \| 5 \| 80.00  (11.60) \| 73.20  (18.10) \| 66.60  (14.90) \| \| |
| Mehl et al. (2016)  4 unspecified minipigs  3 males and 1 female  Unspecified age | maxilla and mandible  all pre-molars and 1^st^ molars  12 weeks | 64 implants  Ø4.3x9mm  Promote^®^ (presumed)  delayed/3months submerged + 6 months not submerged  36 weeks | mineralized  light microscopy  buccal-lingual  toluidine blue  30-40μm | \|  \| Implant surface \| \| --- \| --- \| \| weeks \| Promote \| \| 36 \| 61.00(23.00) \| |
| Ou et al. (2016) a  12 unspecified minipigs  unspecified sex  12 months old | mandible  no extraction  NA (implants placed in edentulous areas between teeth) | 36 implants  Ø4.5x11mm  Machined; Hung Chun Bio-S SLA and SLAffinity  NA/submerged  3 and 6 weeks | mineralized  light microscopy  mesio-distal  toluidine blue  50μm | \|  \| Implant surface \| \| \| \| --- \| --- \| --- \| --- \| \| weeks \| machined \| SLA \| SLAffinity \| \| 3 \| 51.87  (2.14) \| 63.47  (2.45) \| 71.87  (1.97) \| \| 6 \| 73.58  (1.54) \| 77.54  (1.34) \| 80.54  (1.35) \| |
| Ou et al. (2016) b  18 Lanyu small-ear pigs  all males  12 months old | mandible  no extraction  NA (implants placed in edentulous areas between teeth) | 72 implants  Ø4x8mm  Machined; Hung Chun Bio-S SLA and SLAffinity  NA/submerged  2, 4, and 8 weeks | mineralized  light microscopy  mesio-distal  toluidine blue  50μm | \|  \| Implant surface \| \| \| \| --- \| --- \| --- \| --- \| \| weeks \| machined \| SLA \| SLAffinity \| \| 2 \| 17.00  (5.40) \| 20.50  (7.20) \| 28.50  (6.20) \| \| 4 \| 53.80  (6.90) \| 65.10  (5.70) \| 71.30  (7.30) \| \| 8 \| 76.50  (6.30) \| 81.10  (8.40) \| 84.30  8.10) \| |
| Stavropoulos et al. (2016)  11 Göttingen minipigs  all females  30 months old | mandible  2^nd^, 3^rd^, and 4^th^ pre-molars and 1^st^ molars  12 weeks | 44 implants  Ø4.1x8mm  SLActive^®^  delayed/ 1 group submerged and 1 group not submerged  4 and 8 weeks | mineralized  light microscopy  buccal-lingual and mesio-distal  Paragon  30-50 μm | \|  \|  \| Osteotomy protocol \| \| \| --- \| --- \| --- \| --- \| \| weeks \| loading \| Standard \| Drill only \| \| 4 \| no \| 58.40  (12.80) \| 60.70  (19.00) \| \| 8 \| 8 weeks \| 66.00  (13.70) \| 74.80  (11.20) \| |
| Botzenhart et al. (2015)  5 unspecified minipigs  all females  48 months old | maxilla  no extraction  NA (implants placed in edentulous areas between teeth) | 15 implants  Ø4.2x5mm  Dentaurum Implants^®^: ceramic blasted; acid etched and ceramic blasted  NA/submerged  8 weeks | mineralized  light microscopy  unclear cut direction  Masson-Goldner Trichrome  150 μm | \|  \| Implant surface \| \| \| \| --- \| --- \| --- \| --- \| \|  \| ceramic blasted \| acid etched and ceramic blasted \| \| \| weeks \| Tiologic 9mm \| Tiologic  9 mm \| Tiologic 5mm \| \| 8 \| 48.90  (14.80) \| 77.20  (19.80) \| 65.50  (12.00) \| |
| Huang et al. (2015)  6 Lanyu small-ear pigs  unspecified sex  12 months old | mandible  no extraction  NA (implants placed in edentulous areas between teeth) | 12 implants  Ø4.5x11mm  machined and Hung Chun Bio-S SLAffinity  NA/submerged  3 and 6 weeks | mineralized  light microscopy  mesio-distal  toluidine blue  50 μm | \|  \| Implant surface \| \| \| --- \| --- \| --- \| \| weeks \| machined \| SLAffinity \| \| 3 \| 62.61(4.21) \| 71.38(3.14) \| \| 6 \| 72.96(5.18) \| 78.84(3.99) \| |
| López-García et al. (2015)  11 unspecified minipigs  all females  60 months old | maxilla and mandible  3 pre-molars and 1^st^ molars  16 weeks | 120 implants  Ø3.8x12mm  Klockner SK: Gblast and 2Step  delayed/not submerged  24 weeks | mineralized  light microscopy  buccal-lingual  Levai-Laczko  40 μm | \|  \| Implant surface \| \| \| --- \| --- \| --- \| \| weeks \| Gblast \| 2Step \| \| 24 \| 42.73(20.82) \| 72.28(16.68) \| |
| Korn et al. (2014)  6 Berlin minipigs  all females  45 months old | mandible  pre-molars  8 weeks | 36 implants  Ø5x15mm  Thommen Medical^®^ AG- Grit-blasted and thermally acid-etched: uncoated control (TI); collagen/chondroitin sulfate (Coll/CS); collagen/hyaluronan sulfate (Coll/Hya)  delayed/submerged  4 and 8 weeks | mineralized  light microscopy  buccal-lingual  Masson-Goldner Trichrome  100 μm | \|  \| Implant surface \| \| \| \| --- \| --- \| --- \| --- \| \| weeks \| TI \| Coll/CS \| Coll/Hya \| \| 4 \| 44.30  (10.20) \| 47.60  (11.00) \| 37.00  (13.50) \| \| 8 \| 41.20  (10.70) \| 44.70  (11.20) \| 42.70  (9.70) \| |
| Schulz et al. (2014)  6 Berlin minipigs  all females  45 months old | maxilla  pre-molars  8 weeks | 36 implants  Ø5x15mm  Thommen Medical^®^ AG- Grit-blasted and thermally acid-etched: uncoated control (TI); collagen/chondroitin sulfate (Coll/CS); collagen/hyaluronan sulfate (Coll/Hya)  delayed/submerged  4 and 8 weeks | mineralized  light microscopy  buccal-lingual  Masson-Goldner Trichrome  100 μm | \|  \| Implant surface \| \| \| \| --- \| --- \| --- \| --- \| \| weeks \| TI \| Coll/CS \| Coll/Hya \| \| 4 \| 33.30  (11.80) \| 37.00  (17.00) \| 33.10  (14.00) \| \| 8 \| 31.40  (17.60) \| 37.00  (14.80) \| 48.00  (13.50) \| |
| Sivan-Gildor et al. (2014)  3 Sinclair minipigs  all females  12 months old | mandible  1^st^ to 4^th^ pre-molars  NA | 23 implants  Ø6x6mm  Cortex Dental Implants^®^- Molarty (unclear surface description)  immediate/submerged  14 weeks | mineralized  light microscopy  unclear cut direction  hematoxylin-eosin  30 μm | \|  \| Implant design \| \| \| --- \| --- \| --- \| \| weeks \| Standard \| Novel \| \| 14 \| 33.30  (11.80) \| 37.00  (17.00) \| |
| Stramandinoli-Zanicotti et al. (2014)  12 Br1 minipigs  all males  18 months old | mandible  3^rd^ and 4^th^ pre-molars  NA | 96 implants  unclear implant size  Neodent^®^ Neoporos (presumed)  immediate/submerged  13 weeks | mineralized  light microscopy  unclear cut direction  Stevenel’s blue and alizarin red S  20 μm | \|  \| Implant surface \| \| --- \| --- \| \| weeks \| Neoporos \| \| 13 \| 33.10  (18.00) \| |
| Vasak et al. (2014)  9 Göttingen minipigs  all females  20-22 months old | maxilla and mandible  pre-molars  12 weeks | 108 implants  Ø3.5x8mm  Thommen Medical^®^ AG- Control (Sandblasted and hot acid etched); INICELL^®^; SLActive^®^  5, 10, and 15 days | mineralized  light microscopy  buccal-lingual  Levai-Laczko  30 μm | \|  \| Implant surface \| \| \| \| --- \| --- \| --- \| --- \| \| days \| control \| INICELL^®^ \| SLActive^®^ \| \| 5 \| 10.10  (5.20) \| 9.00  (5.30) \| 22.60  (16.20) \| \| 10 \| 56.80  (25.70) \| 57.40  (20.80) \| 68.50  (10.10) \| \| 15 \| 52.40  (16.20) \| 69.70  (12.80) \| 57.40  (14.40) \| |
| Verket et al. (2014)  3 Göttingen minipigs  all females  20-22 months old | mandible  2^nd^ to 4^th^ pre-molars  12 weeks | 30 implants  Ø3.3x10mm  SLActive^®^(presumed)  delayed/submerged  6 weeks | mineralized  light microscopy  buccal-lingual  hematoxylin-eosin  70 μm | \| weeks \| Sham group \| \| --- \| --- \| \| 6 \| 60.90  (31.20) \| |
| Liñares et al. (2013)  6 Göttingen minipigs  all females  14-16 months old | mandible  2^nd^, 3^rd^, and 4^th^ pre-molars and 1^st^ molars  12 weeks | 36 implants  Ø3.3x8mm  TimodSLA (SLActive^®^)  delayed/not submerged  8 weeks | mineralized  light microscopy  mesio-distal  Paragon  80-100 μm | \|  \| Implant collar surface \| \| \| \| --- \| --- \| --- \| --- \| \| weeks \| Ti machined \| Ti modified acid-etched \| Ti alloyed with Zr modified acid-etched \| \| 8 \| 84.24  (3.60)* \| 83.19  (5.70) \| 81.51  (4.50) \| |
| Eom et al. (2012)  12 unspecified minipigs  all males  adults | mandible  1^st^ to 4^th^ pre-molars and 1^st^ molars  16 weeks | 72 implants  Ø3.7x8.5mm  Osstem Implant^®^: RBM (blasting HA); SLA (alumina blasting + dual acid etching); HA (hybrid type coating with HA and RBM)  delayed/unclear healing type  2, 4, and 8 weeks | mineralized  light microscopy  buccal-lingual  hematoxylin-eosin  30-40 μm | \|  \| Implant surface \| \| \| \| --- \| --- \| --- \| --- \| \| weeks \| RBM \| SLA \| HA \| \| 2 \| 46.18  (21.69) \| 51.36  (22.36) \| 65.74  (12.89) \| \| 4 \| 63.38  (23.21) \| 63.79  (12.02) \| 79.24  (10.18) \| \| 8 \| 58.50  (14.98) \| 66.15  (19.02) \| 85.97  (7.29) \| |
| Gahlert et al. (2012)  18 Göttingen minipigs  all females  23.7 months old | maxilla  canines and incisors  24 weeks | 36 implants  Ø4.1x10mm  SLA^®^  delayed/submerged  4, 8, and 12 weeks | mineralized  light microscopy  unclear cut direction  Giemsa-eosin  120 μm | \| weeks \| Ti SLA^®^ (control group) \| \| --- \| --- \| \| 4 \| 64.70(9.40) \| \| 8 \| 79.20(1.70) \| \| 12 \| 83.70(10.30) \| |
| Gottlow et al. (2012)  12 Göttingen minipigs  all females  14-16 months old | mandible  1^st^ to 3^rd^ pre-molar and 1^st^ molars  12 weeks | 72 implants  Ø4.2x5mm  SLActive^®^  delayed/submerged  4 weeks | mineralized  light microscopy  mesio-distal  toluidine-blue  10 μm | \|  \| Implant material \| \| \| --- \| --- \| --- \| \| weeks \| Ti \| TiZr \| \| 4 \| 72.30(20.50) \| 70.20(17.30) \| |
| Saulacic et al. (2012)  12 unspecified minipigs  all females  24 months old | maxilla  6 incisors  12 weeks | 72 implants  Ø4.2x6mm  SLActive^®^; sand-blasted and acid-washed surface  1, 2, 4, and 8 weeks | mineralized  light microscopy  buccal-lingual  toluidine-blue  80 μm | \|  \| Implant surface \| \| \| \| \| \| --- \| --- \| --- \| --- \| --- \| --- \| \|  \| SLActive^®^ \| \| \| sand-blasted and acid-washed \| \| \|  \| Implant material \| \| \| \| \| weeks \| TiZr \| cpTi \| \| Ti6Al4V \| \| 1 \| 9.19  (7.10) \| 13.62  (10.44) \| \| 9.15  (8.21) \| \| 2 \| 59.38  (19.73) \| \| 76.15  (31.62) \| 42.29  (31.41) \| \| 4 \| 74.09  (16.45) \| \| 75.48  (17.69) \| 26.79  (12.87) \| \| 8 \| 74.50  (10.61) \| \| 84.67  (12.62) \| 28.60  (31.52) \| |
| Stadlinger et al. (2012)  20 unspecified minipigs  10 males and 10 females  12 months old | maxilla  deciduous and permanents pre-molars  9 weeks | 60 implants  Ø4.5x9.5mm  Friadent-Dentsply^®^:  Sandblasted with corundum and acid etched (Tit); collagen I coating (Col1); chondroitin sulfate (CS1 and CS2) coatings; sulfated hyaluron coatings (Hya1 and Hya 2)  delayed/submerged  4 and 8 weeks | mineralized  light microscopy  buccal-lingual  Masson-Goldner  30 μm | \|  \| Healing time \| \| \| --- \| --- \| --- \| \| Implant surface \| 4 weeks \| 8 weeks \| \| Tit \| 56.40(13.40)* \| 62.10(13.49) \| \| Col1 \| 42.60(23.13) \| 67.30(11.95) \| \| CS1 \| 68.30(13.85) \| 74.20(11.23) \| \| CS2 \| 51.00(19.17) \| 63.40(10.02) \| \| Hya1 \| 49.50(14.67) \| 63.70(10.73) \| \| Hya2 \| 46.70(13.95) \| 66.30(14.51) \|   *median(SD calculated) |
| Elian et al. (2011)  12 Göttingen minipigs  all females  14-16 months old | mandible  pre-molars and 1^st^ molars  12 weeks | 72 implants  Ø4.1x8mm  SLA^®^  delayed/not submerged  8 weeks | mineralized  light microscopy  buccal-lingual and mesio-distal  Paragon  30 μm | \|  \| Interimplant distance \| \| \| \| --- \| --- \| --- \| --- \| \| weeks \| 2mm \| 3mm \| \| \| 8 \| 76.53(13.27) \| \| 77.92(17.37) \| \| |
| Liñares et al. (2011)  6 Göttingen minipigs  all females  14-16 months old | mandible  2^nd^, 3^rd^, and 4^th^ pre-molars and 1^st^ molars  NA | 24 implants  Ø3.3x8mm  modSLA (SLActive^®^)  immediate/not submerged  8 and 12 weeks | mineralized  light microscopy  buccal-lingual  toluidine blue  70 μm | \|  \| Loading \| \| \| \| \| --- \| --- \| --- \| --- \| --- \| \| weeks \| Immediate \| Delayed \| \| \| \| 8 \| 65.10(6.2) \| \|  \|  \| \| \| 12 \|  \| 66.10(1.3) \| \| \| |
| Ruehe et al. (2011)  5 Göttingen minipigs  unspecified sex  24 months old | maxilla and mandible  all pre-molars and 1^st^ molars  8 weeks | 15 implants  Ø3.5x9mm  OsseoAttract^®^  delayed/submerged  12 weeks | mineralized  light microscopy  mesio-distal  von Kossa  40-60 μm | \| Weeks \| Maxilla \| Mandible \| \| --- \| --- \| --- \| \| 12 \| 52.72(18.54) \| 55.87(9.61) \| \|  \|  \| \| |
| Assenza et al. (2010)  6 Göttingen minipigs  unspecified sex  14-16 months old | mandible  3 pre-molars and 3 molars  12 weeks | 60 implants  Ø4.1x10mm  RF, Bone System - sandblasted and acid etched  delayed/not submerged  12 weeks | mineralized  light microscopy  unclear cut orientation  acid fuchsin and toluidine blue  30 μm | \|  \| Type of restoration \| \| \| \| --- \| --- \| --- \| --- \| \| weeks \| Acrylic \| \| Metallic \| \| \| 12 \| 69.8(3.2) \| 68.1(2.1) \| \| |
| Duyck et al. (2010)  10 Göttingen minipigs  all males  18 months old | maxilla and mandible  4^th^ pre-molars and 1^st^ molars  12 weeks | 80 implants  Ø3.5x8mm  sandblasting and acid etching (experimental) x Osseospeed^®^ (control)  delayed/submerged  4 and 12 weeks | mineralized  light microscopy  buccal-lingual  Stevenel's blue and Von Gieson's picrofuchsin  20-30 μm | \|  \| Implant surface \| \| \| --- \| --- \| --- \| \| weeks \| Experimental \| Control \| \| 8 \| 83.6(1.4) \| 85.9(3.7)5.8(9.2) \| |
| Schliephake et al. (2010)  12 unspecified minipigs  all females  19.1 months old | mandible  deciduous and permanents pre-molars  8 weeks | 72 implants  Ø4.2x8mm  Thommen Medical^®:^ sandblasting and acid etching  delayed/submerged  4 and 13 weeks | mineralized  light microscopy  buccal-lingual  toluidine blue and alizarine-methyleneblue  70 μm | \| weeks \| Ti  sandblasted and acid etched (Group 3 – control) \| \| --- \| --- \| \| 4 \| 69.3(17.1) \| \| 13 \| 78.9(7.23) \| |
| Stadlinger et al. (2010)  7 unspecified minipigs  unspecified sex  72 months old | mandible  deciduous and permanents pre-molars  9 weeks | 21 implants  Ø4x10mm  Friadent-Dentsply^®^:  sandblasting and acid etching  delayed/submerged  4 weeks | mineralized  light microscopy  buccal-lingual  Masson-Goldner  30 μm | \| weeks \| Ti material \| \| --- \| --- \| \| 4 \| 53.08(7.23) \| |
| Stadlinger et al. (2009) a  18 Mini-Lewe minipigs  all females  18 months old | mandible  deciduous and permanents pre-molars  9 weeks | 144 implants  Ø3.5x9.5mm  Thommen Medical^®:^ sandblasted and thermally acid etching without (control) or with (conSF) conditioning with hydroxide ions  delayed/submerged  2, 4, and 8 weeks | mineralized  light microscopy  buccal-lingual  Masson-Goldner  30 μm | \| weeks \| Control \| conSF \| \| --- \| --- \| --- \| \| 2 \| 48.05**  (14.49) \| 65.49  (12.15) \| \| 4 \| 67.40  (12.74) \| 65.73  (12.97) \| \| 8 \| 64.00  (11.83) \| 70.21  (14.85) \|   **median(calculated SE) |
| Stadlinger et al. (2009) b  20 unspecified minipigs  females and castrated males  12 months old | mandible  pre-molars  9 weeks | 60 implants  Ø4.5x9.5mm  Friadent-Dentsply^®^:  sandblasted with corundum (control); coating collagen with low CS content (CS1); coating collagen with high CS content (CS2)  delayed/submerged  4 and 8 weeks | mineralized  light microscopy  buccal-lingual  Masson-Goldner  30 μm | \|  \| Implant surface \| \| \| \| --- \| --- \| --- \| --- \| \| weeks \| Control \| CS1 \| CS2 \| \| 4 \| 51.60  (10.41) \| 68.40  (10.25) \| 63.10  (10.89) \| \| 8 \| 62.70  (10.73) \| 71.00  (10.81) \| 67.90  (10.73) \|   (SD calculated) |
| Traini et al. (2009)  5 Göttingen minipigs  unspecified sex  18-21 months old | mandible  pre-molars and 1^st^ molars  12 weeks | 25 implants  Ø3.8x11mm  Friadent^®^ (unclear surface description)  delayed/not submerged  16 weeks | mineralized  back scattered SEM  buccal-lingual  NA  0.5 μm | \|  \| Loading \| \| \| --- \| --- \| --- \| \| weeks \| Not loaded \| Immediately loaded \| \| 16 \| 78.00  (5.80) \| 77.80  (5.90) \| |
| Stadlinger et al. (2008) a  12 unspecified minipigs  unspecified sex  12 months old | mandible  deciduous and permanents pre-molars  12 weeks | 60 implants  Ø4x13mm  Friadent^®^: sandblasted with corundum (control uncoated); coating collagen typeI; coating collagen type I + collagen type III; RGD-peptide; mineralized collagen type I  delayed/not submerged  24 weeks | mineralized  light microscopy  unclear cut direction  Masson-Goldner  30 μm | \|  \| Healing time \| \| \| --- \| --- \| --- \| \| Implant surface \| 24 weeks \| \| Uncoated \| 60.05(6.27)* \| \| CollagenI \| 62.20(6.10) \| \| CollagenI + III \| 62.20(6.10) \| \| RGD peptide \| 57.00(6.45) \| \| Mineralized collagen \| 55.00(10.78) \|   (SD calculated) |
| Stadlinger et al. (2008) b  20 unspecified minipigs  10 males and 10 females  12 months old | mandible  deciduous and permanents pre-molars  32 weeks | 120 implants  Ø4.25x12mm  Friadent^®^: sandblasted with corundum with coatings: Collagen (Coll); Collagen/Condroitin sulfate (Coll/CS); Collagen/CS/rhBMP-4 (Coll/CS/BMP)  delayed/not submerged  24 weeks | mineralized  light microscopy  unclear cut direction  Masson-Goldner  30 μm | \|  \| Implant surface \| \| \| \| --- \| --- \| --- \| --- \| \| weeks \| Coll \| Coll/CS \| Coll/CS/BMP \| \| 24 \| 40.00  (15.97) \| 30.00  (15.97) \| 27.00  (14.83) \|   (SD calculated) |
| Germanier et al. (2006)  6 unspecified minipigs  unspecified sex  adults | maxilla  anterior teeth  24 weeks | 48 implants  Ø4.2x6mm  SLA^®^; poly-ethylene glycol (PEG); Arg-Asp-Gly (RDG); Arg-Gly-Asp (RGD)  delayed/submerged  2 and 4 weeks | mineralized  light microscopy  buccal-lingual  toluidine-blue + basic fuchsin  80 μm | \|  \| Healing time \| \| \| --- \| --- \| --- \| \| Implant surface \| 2 weeks \| 4 weeks \| \| SLA^®^ \| 43.62(10.79) \| 62.46(6.37) \| \| PEG \| 55.94(8.46) \| 67.40(9.04) \| \| RDG \| 48.54(8.27) \| 75.54(5.93) \| \| RGD \| 61.68(4.21) \| 62.52(8.04) \| |
| Nkenke et al. (2005)  9 Göttingen minipigs  all females  unspecified age | maxilla  3 pre-molars and 1^st^ molars  12 weeks | 108 implants  Ø3.8x13mm  Friadent^®^  delayed/submerged  24 weeks | mineralized  light microscopy  buccal-lingual  toluidine-blue  20 μm | \| Implant site preparation technique/healing period before loading (months) \| buccal \| palatal \| \| --- \| --- \| --- \| \| Osteotome \|  \|  \| \| 0 \| 82(7) \| 79(7) \| \| 1-3 \| 87(10) \| 82(24) \| \| 4-5 \| 72(22) \| 75(16) \| \| Spiral drills \|  \|  \| \| 0 \| 79(6) \| 59(30) \| \| 1-3 \| 58(31) \| 53(26) \| \| 4-5 \| 77(22) \| 76(18) \| |
| Rimondini et al. (2005)  8 unspecified minipigs  all males  adults | mandible  pre-molars  NA | 16 implants  Ø4.5x13mm  Friadent^®^  immediate/not submerged  7, 15, 30, and 60 days | mineralized  light microscopy  buccal-lingual  fast green, toluidine-blue and acid-fuchsin  70-80 μm | \|  \| \| Implant third \| \| \| \| --- \| --- \| --- \| --- \| --- \| \| days \| \| Coronal \| \| Middle \| Apical \| \| 7 \| \| 0.54  (0.0) \| \| 11.28  (18.64) \| 53.45  (37.34) \| \| 15 \| \| 17.2  (8.41) \| \| 22.41  (17.45) \| 41.45  (13.03) \| \| 30 \| \| 43.4  (26.72) \| \| 35.16  (10.33) \| 27.92  (22.05) \| \| 60 \| \| 69.19  (34.78) \| \| 47.38  (31.37) \| 67.38  (35.16) \| |
| Buser et al. (2004)  6 unspecified minipigs  unspecified sex  adults | maxilla  anterior teeth  24 weeks | 48 implants  Ø4.2x6mm  SLA standard (SLA^®^); SLAmodified (SLActive^®^)  delayed/submerged  2, 4, and 8 weeks | mineralized  light microscopy  buccal-lingual  toluidine-blue  80 μm | \| weeks \| SLA standard \| SLA modified \| \| --- \| --- \| --- \| \| 2 \| 29.42  (7.58) \| 49.30  (7.49) \| \| 4 \| 66.57  (8.14) \| 81.91  (3.59) \| \| 8 \| 75.45  (7.66) \| 78.47  (11.14) \| |
| Nkenke et al. (2003)  7 Göttingen minipigs  all females  18-21 months old | mandible  1^st^ to 3^rd^ pre-molars and 1^st^ molar left side  12 weeks | 35 implants  Ø3.8x11mm  Friadent^®^ (presumed)  delayed/ 1 group submerged and 1 group not submerged  16 weeks | mineralized  light microscopy  buccal-lingual  toluidine-blue  20 μm | \|  \| Loading \| \| \| --- \| --- \| --- \| \| weeks \| Not loaded \| Immediately loaded \| \| 16 \| 78.00  (5.80) \| 77.80  (5.90) \| |
| Zechner et al. (2003)  12 hybrids of Minnesota and Vietnamese pot-bellied pigs  unspecified sex  adults | mandible  1^st^ to 3^rd^ pre-molars  NA | 72 implants  Ø3.75x10mm  Machined; HA coated; TiUnite^®^  immediate/submerged  3, 6, and 12 weeks | mineralized  light microscopy  buccal-lingual  Levai-Laczko  20μm | \|  \| \| Implant surface \| \| \| \| --- \| --- \| --- \| --- \| --- \| \| weeks \| \| machined \| \| HA coated \| anodized  (TiUnite^®^) \| \| 3 \| \| 13.04  (7.32) \| \| 25.66  (7.32) \| 26.87  (7.32) \| \| 6 \| \| 17.60  (7.05) \| \| 44.30  (8.85) \| 40.73  (7.05) \| \| 12 \| \| 29.88  (7.12) \| \| 48.49  (6.58) \| 61.29  (6.61) \| |
| Dostálová et al. (2001)  4 unspecified minipigs  unspecified sex  24 months old | mandible  pre-molars  10 weeks | 24 implants  Ø 3.3x12mm  Sand-blasted without (Titanium) or with hydroxilapatite coating (HA coated)  delayed/submerged for 16 weeks and not submerged for  24 weeks  40 weeks | mineralized  light microscopy  unclear cut direction  stain not reported  100μm | \|  \| \| Implant surface \| \| \| --- \| --- \| --- \| --- \| \| weeks \| \| Titanium  (Not coated) \| \| HA coated \| \| 40 \| \| 65.20  (13.5) \| \| 77.50  (10.20) \| |
| Basquill et al. (1994)  9 unspecified minipigs  unspecified sex  unspecified age | mandible  pre-molars  10 weeks | 54 implants  Ø3.75x10mm  Nobelpharma - unclear surface description  delayed/submerged  14 weeks | mineralized  light microscopy  buccal-lingual  toluidine-blue  75 μm | \| weeks \| Control (no radiation) \| \| --- \| --- \| \| 14 \| 42.58(19.00) \|   (Calculated based on Table 4) |

Ti=Titanium. Zr=zirconium.

Anthogyr^®^ - grit blasted with biphasic calcium phosphate and acid etched

Biotech Dental Kontact N^®^ - grit blasted with biphasic calcium phosphate ceramic particles and acid-etched

Camlog Promote^®^ - abrasive-blasted, acid etched

ComMed – SLA sand-blasted large grit acid-etched

Dentsply Astra Osseospeed^®^ - fluoride-treated nanostructured; Friadent^®^ - grit blasted and thermally acid etched

DOT GmbH SA – corundum blasted and acid-etched; BONIT^®^ - CaP coated (HA blasted + double acid etching)

FORESTADENT^®^ - OsseoAttract- acid-etching

Klockner SA ContactTi^®^ - alumina particle bombardment of titanium, bioactivated when treated thermochemically; Shotblasting - shotblasted with alumina particles and acid attack with hydrochloric acid

Klockner SK Gblast – grit-blasted with alumina particles; 2Step – grit-blasted and alkaline-etched

Nobel Biocare TiUnite^®^ - anodized; TiUltra^®^ - gradually anodized with protective layer

Neodent NeoPoros^®^ - sandblasted and acid etched

Straumann SLA^®^ - sandblasted and acid etched; SLActive^®^ - sandblasted and acid etched under a nitrogen atmosphere

Thommen Medical Inicell^®^ - superhydrophilic surface as a result of conditioning with APLIQUIQ^®^ syste
