# Supplementary material for: The minipig intraoral dental implant model: A systematic review and meta-analysis
Source: PLoS One. 2022 Feb 28;17(2):e0264475. doi: 10.1371/journal.pone.0264475 (PMC8884544; doi:10.1371/journal.pone.0264475)
Supplement: S4 Table — (DOCX) [file pone.0264475.s005.docx]

Supplemental Table 4: Quality assessment of included studies (based on ARRIVE checklist)

| Study  Author (year) | Selected items from ARRIVE | | | | | | | | | | |
| --- | --- | --- | --- | --- | --- | --- | --- | --- | --- | --- | --- |
|  | # Groups | Allocation to  treatment | Masking/ blinding | Calibration | Unit of analysis | Surgical  protocol | Animal description | Housing,husbandry and welfare | Sample size | Animal loss | Implant loss |
| Kammerer et al. (2020) |  |  |  |  |  |  |  |  |  |  |  |
| Hoornaert et al. (2020) |  |  |  |  |  |  |  |  |  |  |  |
| Karl et al. (2020) |  |  |  |  |  |  |  |  |  |  |  |
| Thome et al. (2020) |  |  |  |  |  |  |  |  |  |  |  |
| Romero-Ruiz et al. (2019) |  |  |  |  |  |  |  |  |  |  |  |
| Susin et al. (2019) a |  |  |  |  |  |  |  |  |  |  |  |
| Susin et al. (2019) b |  |  |  |  |  |  |  |  |  |  |  |
| Herrero-Climent et al. (2018) |  |  |  |  |  |  |  |  |  |  |  |
| Hou et al. (2018) |  |  |  |  |  |  |  |  |  |  |  |
| Mehl et al. (2018) |  |  |  |  |  |  |  |  |  |  |  |
| Rios-Santos et al. (2018) |  |  |  |  |  |  |  |  |  |  |  |
| Kuo et al. (2017) |  |  |  |  |  |  |  |  |  |  |  |
| Brockemeyer et al. 2016 |  |  |  |  |  |  |  |  |  |  |  |
| Chiang et al. (2016) |  |  |  |  |  |  |  |  |  |  |  |
| Cochran et al. 2016 |  |  |  |  |  |  |  |  |  |  |  |
| Eom et al. 2016 |  |  |  |  |  |  |  |  |  |  |  |
| Mehl et al. 2016 |  |  |  |  |  |  |  |  |  |  |  |
| Ou et al. (2016) a |  |  |  |  |  |  |  |  |  |  |  |
| Ou et al. (2016) b |  |  |  |  |  |  |  |  |  |  |  |
| Stavropoulos et al. (2016) |  |  |  |  |  |  |  |  |  |  |  |
| Botzenhart et al. (2015) |  |  |  |  |  |  |  |  |  |  |  |
| Huang et al. (2015) |  |  |  |  |  |  |  |  |  |  |  |
| López García et al. (2015) |  |  |  |  |  |  |  |  |  |  |  |
| Korn et al. (2014) |  |  |  |  |  |  |  |  |  |  |  |
| Schulz et al. (2014) |  |  |  |  |  |  |  |  |  |  |  |
| Sivan-Gildor et al. (2014) |  |  |  |  |  |  |  |  |  |  |  |
| Stramandinolli-Zanicotti et al. (2014) |  |  |  |  |  |  |  |  |  |  |  |
| Vasak et al. (2014) |  |  |  |  |  |  |  |  |  |  |  |
| Verket et al. (2014) |  |  |  |  |  |  |  |  |  |  |  |
| Liñares et al. (2013) |  |  |  |  |  |  |  |  |  |  |  |
| Eom et al. (2012) |  |  |  |  |  |  |  |  |  |  |  |
| Gahlert et al. (2012) |  |  |  |  |  |  |  |  |  |  |  |
| Gottlow et al. (2012) |  |  |  |  |  |  |  |  |  |  |  |
| Saulacic et al. (2012) |  |  |  |  |  |  |  |  |  |  |  |
| Stadlinger et al. (2012) |  |  |  |  |  |  |  |  |  |  |  |
| Elian et al. (2011) |  |  |  |  |  |  |  |  |  |  |  |
| Linares et al. (2011) |  |  |  |  |  |  |  |  |  |  |  |
| Ruehe et al. (2011) |  |  |  |  |  |  |  |  |  |  |  |
| Assenza et al. (2010) |  |  |  |  |  |  |  |  |  |  |  |
| Duyck et al. (2010) |  |  |  |  |  |  |  |  |  |  |  |
| Schliephake et al. (2010) |  |  |  |  |  |  |  |  |  |  |  |
| Stadlinger et al. (2010) |  |  |  |  |  |  |  |  |  |  |  |
| Stadlinger et al. (2009)a |  |  |  |  |  |  |  |  |  |  |  |
| Stadlinger et al. (2009)b |  |  |  |  |  |  |  |  |  |  |  |
| Traini et al. (2009) |  |  |  |  |  |  |  |  |  |  |  |
| Stadlinger et al. (2008)a |  |  |  |  |  |  |  |  |  |  |  |
| Stadlinger et al. (2008)b |  |  |  |  |  |  |  |  |  |  |  |
| Germanier et al. (2006) |  |  |  |  |  |  |  |  |  |  |  |
| Nkenke et al. (2005) |  |  |  |  |  |  |  |  |  |  |  |
| Rimondini et al. (2005) |  |  |  |  |  |  |  |  |  |  |  |
| Buser et al. (2004) |  |  |  |  |  |  |  |  |  |  |  |
| Nkenke et al. (2003) |  |  |  |  |  |  |  |  |  |  |  |
| Zechner et al. (2003) |  |  |  |  |  |  |  |  |  |  |  |
| Dostalova et al. (2001) |  |  |  |  |  |  |  |  |  |  |  |
| Basquill et al. (1994) |  |  |  |  |  |  |  |  |  |  |  |
